# Strong-Separation Logic

**DOI:** 10.1007/978-3-030-72019-3_24

**Published:** 2021-03-23

**Authors:** Jens Pagel, Florian Zuleger

**Affiliations:** Imperial College, London, UK; TU Wien, Vienna, Austria

## Abstract

Most automated verifiers for separation logic are based on the symbolic-heap fragment, which disallows both the magic-wand operator and the application of classical Boolean operators to spatial formulas. This is not surprising, as support for the magic wand quickly leads to undecidability, especially when combined with inductive predicates for reasoning about data structures. To circumvent these undecidability results, we propose assigning a more restrictive semantics to the separating conjunction. We argue that the resulting logic, strong-separation logic, can be used for symbolic execution and abductive reasoning just like “standard” separation logic, while remaining decidable even in the presence of both the magic wand and the list-segment predicate—a combination of features that leads to undecidability for the standard semantics.

### 1 Introduction

Separation logic [40] is one of the most successful formalisms for the analysis and verification of programs making use of dynamic resources such as heap memory and access permissions [7,30,10,5,17,24,9]. At the heart of the success of separation logic (SL) is the *separating conjunction*, $*$, which supports concise statements about the disjointness of resources. In this article, we will focus on separation logic for describing the heap in single-threaded heap-manipulating programs. In this setting, the formula $\varphi * \psi$ can be read as "the heap can be split into two disjoint parts, such that $\varphi$ holds for one part and $\psi$ for the other."

Our article starts from the following observation: The standard semantics of $*$ allows splitting a heap into two arbitrary sub-heaps. The magic-wand operator $-\!\!*$, which is the adjoint of $*$, then allows adding arbitrary heaps. This arbitrary splitting and adding of heaps makes reasoning about SL formulas difficult, and quickly renders separation logic undecidable when inductive predicates for data structures are considered. For example, Demri et al. recently showed that adding only the singly-linked list-segment predicate to propositional separation logic (i.e., with $*, -\!\!*$ and classical connectives $\wedge, \vee, \neg$) leads to undecidability [16].

Most SL specifications used in automated verification do not, however, make use of arbitrary heap compositions. For example, the widely used symbolic-heap fragments of separation logic considered, e.g., in [3,4,13,21,22], have the following property: a symbolic heap satisfies a separating conjunction, if and only if one can split the model at locations that are the values of some program variables.

Motivated by this observation, we propose a more restrictive separating conjunction that allows splitting the heap only at location that are the values of some

(a) A model of $\texttt{ls}(x, y) * \texttt{ls}(y, \mathsf{nil})$ in both the standard semantics and our semantics.

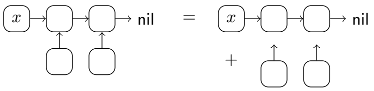

(b) A model of $\texttt{ls}(x, \mathsf{nil}) * \texttt{t}$ in the standard semantics.

Fig. 1: Two models and their decomposition into disjoint submodels. Dangling arrows represent dangling pointers.

program variables. We call the resulting logic *strong-separation logic.* Strong-separation logic (SSL) shares many properties with standard separation-logic semantics; for example, the models of our logic form a separation algebra. Because the *frame rule* and other standard SL inference rules continue to hold for SSL, SSL is suitable for deductive Hoare-style verification à la [23,40], symbolic execution [4], as well as abductive reasoning [10,9]. At the same time, SSL has much better computational properties than standard SL—especially when formulas contain expressive features such as the *magic wand*, $\mathrel{-\!\!*}$, or negation.

We now give a more detailed introduction to the contributions of this article.

*The standard semantics of the separating conjunction.* To be able to justify our changed semantics of $*$, we need to introduce a bit of terminology. As standard in separation logic, we interpret SL formulas over *stack–heap pairs*. A *stack* is a mapping of the program variables to memory locations. A *heap* is a finite partial function between memory locations; if a memory location $l$ is mapped to location $l'$, we say the heap contains a *pointer* from $l$ to $l'$. A memory location $l$ is *allocated* if there is a pointer of the heap from $l$ to some location $l'$. We call a location *dangling* if it is the target of a pointer but not allocated; a pointer is dangling if its target location is dangling.

Dangling pointers arise naturally in compositional specifications, i.e., in formulas that employ the separating conjunction $*$: In the standard semantics of separation logic, a stack–heap pair $(s, h)$ satisfies a formula $\varphi * \psi$, if it is possible to split the heap $h$ into two disjoint parts $h_1$ and $h_2$ such that $(s, h_1)$ satisfies $\varphi$ and $(s, h_2)$ satisfies $\psi$. Here, disjoint means that the allocated locations of $h_1$ and $h_2$ are disjoint; however, the targets of the pointers of $h_1$ and $h_2$ do not have to be disjoint.

We illustrate this in Fig. 1a, where we show a graphical representation of a stack–heap pair $(s, h)$ that satisfies the formula $\texttt{ls}(x, y) * \texttt{ls}(y, \mathsf{nil})$. Here, $\texttt{ls}$ denotes the list-segment predicate. As shown in Fig. 1a, $h$ can be split into two disjoint parts $h_1$ and $h_2$ such that $(s, h_1)$ is a model of $\texttt{ls}(x, y)$ and $(s, h_2)$ is a model of $\texttt{ls}(y, \mathsf{nil})$. Now, $h_1$ has a dangling pointer with target $s(y)$ (displayed with an orange background), while no pointer is dangling in the heap $h$.

*In what sense is the standard semantics too permissive?* The standard semantics of $*$ allows splitting a heap into two arbitrary sub-heaps, which may result in the introduction of arbitrary dangling pointers into the sub-heaps. We note, however, that the introduction of dangling pointers is *not* arbitrary when splitting the models of $\texttt{ls}(x,y) * \texttt{ls}(y,\mathsf{nil})$; there is only one way of splitting the models of this formula, namely at the location of program variable $y$. The formula $\texttt{ls}(x,y)* \texttt{ls}(y,\mathsf{nil})$ belongs to a certain variant of the symbolic-heap fragment of separation logic, and all formulas of this fragment have the property that their models can only be split at locations that are the values of some variables.

Standard SL semantics also allows the introduction of dangling pointers without the use of variables. Fig. 1b shows a model of $\texttt{ls}(x,\mathsf{nil}) * \texttt{t}$—assuming the standard semantics. Here, the formula $\texttt{t}$ (for *true*) stands for any arbitrary heap. In particular, this includes heaps with arbitrary dangling pointers into the list segment $\texttt{ls}(x,\mathsf{nil})$. This power of introducing arbitrary dangling pointers is what is used by Demri et al. for their undecidability proof of propositional separation logic with the singly-linked list-segment predicate [16].

*Strong-separation logic.* In this article, we want to explicitly *disallow* the *implicit* sharing of dangling locations when composing heaps. We propose to parameterize the separating conjunction by the stack and exclusively allow the union of heaps that only share locations that are pointed to by the stack. For example, the model in Fig. 1b is *not* a model of $\texttt{ls}(x,\mathsf{nil}) * \texttt{t}$ in our semantics because of the dangling pointers in the sub-heap that satisfies $\texttt{t}$. *Strong-separation logic* (SSL) is the logic resulting from this restricted definition of the separating conjunction.

*Why should I care?* We argue that SSL is a promising proposal for automated program verification:

1) We show that the memory models of strong-separation logic form a *separation algebra* [11], which guarantees the soundness of the standard *frame rule* of SL [40]. Consequently, SSL can be potentially be used instead of standard SL in a wide variety of (semi-)automated analyzers and verifiers, including Hoare-style verification [40], symbolic execution [4], and bi-abductive shape analysis [10].

2) To date, most automated reasoners for separation logic have been developed for *symbolic-heap separation logic* [3,4,10,21,22,26,32,27]. In these fragments of separation logic, assertions about the heap can exclusively be combined via $*$; neither the magic wand $-\!\!*$ nor classical Boolean connectives are permitted. We show that the strong semantics agrees with the standard semantics on symbolic heaps. For this reason, symbolic-heap SL specifications remain unchanged when switching to strong-separation logic.

3) We establish that the satisfiability and entailment problem for full propositional separation logic with the singly-linked list-segment predicate is decidable in our semantics (in PSPACE)—in stark contrast to the aforementioned undecidability result obtained by Demri et al. [16] assuming the standard semantics.

4) The standard Hoare-style approach to verification requires discharging verification conditions (VCs), which amounts to proving for loop-free pieces of code that a pre-condition implies some post-condition. Discharging VCs can be automated by calculi that symbolically execute the pre-condition forward resp. the post-condition backward, and then using an entailment checker for proving the implication. For SL, symbolic execution calculi can be formulated using the magic wand resp. the septraction operator. However, these operators have proven to be difficult for automated procedures: "VC-generators do not work especially well with separation logic, as they introduce magic-wand $-\!\!*$ operators which are difficult to eliminate." [2, p. 131] In contrast, we demonstrate that SSL can overcome the described difficulties. We formulate a forward symbolic execution calculus for a simple heap-manipulating programming language using SSL. In conjunction with our entailment checker, see 3), our calculus gives rise to a fully-automated procedure for discharging VCs of loop-free code segments.

5) Computing solutions to the *abduction problem* is an integral building block of Facebook's Infer analyzer [9], required for a scalable and fully-automated shape analysis [10]. We show how to compute explicit representations of optimal, i.e., *logically weakest* and *spatially minimal*, solutions to the abduction problem for the separation logic considered in this paper. The result is of theoretical interest, as explicit representations for optimal solutions to the abduction problem are hard to obtain [10,19].

*Contributions.* Our main contributions are as follows:

1. We propose and motivate *strong-separation logic* (SSL), a new semantics for separation logic.
2. We present a PSpace decision procedure for strong-separation logic with points-to assertions, the list-segment predicate $\mathtt{ls}(x, y)$, and spatial and classical operators, i.e., $*$, $-\!\!*$, $\wedge$, $\vee$, $\neg$[1]—a logic that is undecidable when assuming the standard semantics [16].
3. We present symbolic execution rules for SSL, which allow us to discharge verification conditions fully automatically.
4. We show how to compute explicit representations of optimal solutions to the abduction problem for the SSL considered in (2).

We strongly believe that these results motivate further research on SSL (e.g., going beyond the singly-linked list-segment predicate, implementing our decision procedure and integrating it into fully-automated analyzers).

*Related work.* The undecidability of separation logic was established already in [12]. Since then, decision problems for a large number of fragments and variants of separation logic have been studied. Most of this work has been on symbolic-heap separation logic or other variants of the logic that neither support the magic wand nor the use of negation below the $*$ operator. While entailment in the symbolic-heap fragment with inductive definitions is undecidable in general [1], there are decision procedures for variants with built-in lists and/or trees [3,13,34,35,36], support for defining variants of linear structures [20] or tree structures [42,22] or graphs of bounded tree width [21,26]. The expressive heap logics Strand [29] and Dryad [37] also have decidable fragments, as have some other separation logics that allow combining shape and data constraints. Besides the already mentioned work [35,36], these include [28,25].

[1] An extension of this result to a separation logic that also supports trees can be found in the dissertation of the first author [31]

Among the aforementioned works, the graph-based decision procedures of [13] and [25] are most closely related to our approach. Note however, that neither of these works supports reasoning about magic wands or negation below the separating conjunction.

In contrast to symbolic-heap SL, separation logics with the *magic wand* quickly become undecidable. Propositional separation logic with the magic wand, but without inductive data structures, was shown to be decidable in PSpace in the early days of SL research [12]. Support for this fragment was added to CVC4 a few years ago [39]. Some tools have “lightweight” support for the magic wand involving heuristics and user annotations, in part motivated by the lack of decision procedures [6,41].

There is a significant body of work studying first-order SL with the magic wand and unary points-to assertions, but without a list predicate. This logic was first shown to be undecidable in [8]; a result that has since been refined, showing e.g. that while satisfiability is still in PSpace if we allow one quantified variable [15], two variables already lead to undecidability, even without the separating conjunction [14]. Echenim et al. [18] have recently addressed the satisfiability problem of SL with $\exists^*\forall^*$ quantifier prefix, separating conjunction, magic wand, and full Boolean closure, but no inductive definitions. The logic was shown to be undecidable in general (contradicting an earlier claim [38]), but decidable in PSpace under certain restrictions.

*Outline.* In Section 2, we introduce two semantics of propositional separation logic, the standard semantics and our new *strong-separation* semantics. We show the decidability of the satisfiability and entailment problems of SSL with lists in Section 3. We present symbolic execution rules for SSL in Section 4. We show how to compute explicit representations of optimal solutions to the abduction problem in Section 5. We conclude in Section 6. All missing proofs are given in the extended version [33] for space reasons.

## 2 Strong- and Weak-Separation Logic

### 2.1 Preliminaries

We denote by $|X|$ the cardinality of the set $X$. Let $f$ be a (partial) function. Then $\mathrm{dom}(f)$ and $\mathrm{img}(f)$ denote the domain and image of $f$, respectively. We write $|f| := |\mathrm{dom}(f)|$ and $f(x) = \bot$ for $x \notin \mathrm{dom}(f)$. We frequently use set notation to define and reason about partial functions: $f := \{x_1 \mapsto y_1, \ldots, x_k \mapsto y_k\}$ is the partial function that maps $x_i$ to $y_i$, $1 \le i \le k$, and is undefined on all other values; $f^{-1}(b)$ is the set of all elements $a$ with $f(a) = b$; we write $f \cup g$ resp. $f \cap g$ for the union resp. intersection of partial functions $f$ and $g$, provided that $f(a) = g(a)$ for all $a \in \mathrm{dom}(f) \cap \mathrm{dom}(g)$; similarly, $f \subseteq g$ holds if $\mathrm{dom}(f) \subseteq \mathrm{dom}(g)$. Sets and ordered sequences are denoted in boldface, e.g., $\mathbf{x}$. To list the elements of a sequence, we write $\langle x_1, \ldots, x_k \rangle$.

We assume a linearly-ordered infinite set of variables $\mathbf{Var}$ with $\mathsf{nil} \in \mathbf{Var}$ and denote by $\max(\mathbf{v})$ the maximal variable among a set of variables $\mathbf{v}$ according

$$\tau ::= \mathbf{emp} \mid x \mapsto y \mid \mathtt{ls}(x,y) \mid x = y \mid x \neq y$$
$$\varphi ::= \tau \mid \varphi * \varphi \mid \varphi \text{-}\!\circledast \varphi \mid \varphi \wedge \varphi \mid \varphi \vee \varphi \mid \neg\varphi$$

Fig. 2: The syntax of separation logic with list segments.

| | |
|---|---|
| $(s,h) \models \mathbf{emp}$ | iff $\mathrm{dom}(h) = \emptyset$ |
| $(s,h) \models x = y$ | iff $\mathrm{dom}(h) = \emptyset$ and $s(x) = s(y)$ |
| $(s,h) \models x \neq y$ | iff $\mathrm{dom}(h) = \emptyset$ and $s(x) \neq s(y)$ |
| $(s,h) \models x \mapsto y$ | iff $h = \{s(x) \mapsto s(y)\}$ |
| $(s,h) \models \mathtt{ls}(x,y)$ | iff $\mathrm{dom}(h) = \emptyset$ and $s(x) = s(y)$ or there exist $n \geq 1, \ell_0, \ldots, \ell_n$ with $h = \{\ell_0 \mapsto \ell_1, \ldots, \ell_{n-1} \mapsto \ell_n\}, s(x) = \ell_0$ and $s(y) = \ell_n$ |
| $(s,h) \models \varphi_1 \wedge \varphi_2$ | iff $(s,h) \models \varphi_1$ and $(s,h) \models \varphi_2$ |
| $(s,h) \models \neg\varphi$ | iff $(s,h) \not\models \varphi$ |
| $(s,h) \models^{\mathsf{wk}} \varphi_1 * \varphi_2$ | iff there exist $h_1, h_2$ with $h = h_1 + h_2, (s,h_1) \models^{\mathsf{wk}} \varphi_1, (s,h_2) \models^{\mathsf{wk}} \varphi_2$ |
| $(s,h) \models^{\mathsf{wk}} \varphi_1 \text{-}\!\circledast \varphi_2$ | iff exist $h_1$ with $(s,h_1) \models^{\mathsf{wk}} \varphi_1, h + h_1 \neq \bot$ and $(s, h + h_1) \models^{\mathsf{wk}} \varphi_2$ |
| $(s,h) \models^{\mathsf{st}} \varphi_1 * \varphi_2$ | iff there exists $h_1, h_2$ with $h = h_1 \uplus^s h_2, (s,h_1) \models^{\mathsf{st}} \varphi_1, (s,h_2) \models^{\mathsf{st}} \varphi_2$ |
| $(s,h) \models^{\mathsf{st}} \varphi_1 \text{-}\!\circledast \varphi_2$ | iff exists $h_1$ with $(s,h_1) \models^{\mathsf{st}} \varphi_1, h \uplus^s h_1 \neq \bot$ and $(s, h \uplus^s h_1) \models^{\mathsf{st}} \varphi_2$ |

Fig. 3: The standard, "weak" semantics of separation logic, $\models^{\mathsf{wk}}$, and the "strong" semantics, $\models^{\mathsf{st}}$. We write $\models$ when there is no difference between $\models^{\mathsf{wk}}$ and $\models^{\mathsf{st}}$.

to this order. In Fig. 2, we define the syntax of the separation-logic fragment we study in this article. The atomic formulas of our logic are the *empty-heap predicate* **emp**, *points-to assertions* $x \mapsto y$, the *list-segment predicate* $\mathtt{ls}(x,y)$, equalities $x = y$ and disequalities $x \neq y$[2] ; in all these cases, $x, y \in \mathbf{Var}$. Formulas are closed under the classical Boolean operators $\wedge, \vee, \neg$ as well as under the *separating conjunction* $*$ and the existential magic wand, also called the *septraction*, $\text{-}\!\circledast$ (see e.g. [8]). We collect the set of all SL formulas in **SL**. We also consider derived operators and formulas, in particular the *separating implication* (or *magic wand*), $\text{-}\!*$, defined by $\varphi \text{-}\!* \psi := \neg(\varphi \text{-}\!\circledast \neg\psi)$.[3] We also use *true*, defined as $\mathtt{t} := \mathbf{emp} \vee \neg\mathbf{emp}$. Finally, for $\Phi = \{\varphi_1, \ldots, \varphi_n\}$, we define $\bigstar\Phi := \varphi_1 * \varphi_2 * \cdots * \varphi_n$ if $n > 1$ and $\bigstar\Phi := \mathbf{emp}$ if $n = 0$. By $\mathsf{fvs}(\varphi)$ we denote the set of (free) variables of $\varphi$. We define the *size* of the formula $\varphi$ as $|\varphi| = 1$ for atomic formulas $\varphi$, $|\varphi_1 \times \varphi_2| := |\varphi_1| + |\varphi_2| + 1$ for $\times \in \{\wedge, \vee, *, \text{-}\!\circledast\}$ and $|\neg\varphi_1| := |\varphi_1| + 1$.

### 2.2 Two Semantics of Separation Logic

*Memory model.* **Loc** is an infinite set of *heap locations.* A *stack* is a partial function $s\colon \mathbf{Var} \rightharpoonup \mathbf{Loc}$. A *heap* is a partial function $h\colon \mathbf{Loc} \rightharpoonup \mathbf{Loc}$. A *model* is a stack–heap pair $(s,h)$ with $\mathsf{nil} \in \mathrm{dom}(s)$ and $s(\mathsf{nil}) \notin \mathrm{dom}(h)$. We let $\mathsf{locs}(h) :=$

2 As our logic contains negation, $x \neq y$ can be expressed as $\neg(x = y)$. However, we treat disequalities as atomic to be able to use them in the positive fragment of our logic, defined later, which precludes the use of negation.

3 As $\text{-}\!*$ can be defined via $\text{-}\!\circledast$ and $\neg$ and vice-versa, the expressivity of our logic does not depend on which operator we choose. We have chosen $\text{-}\!\circledast$ because we can include this operator in the positive fragment considered later on.

$\mathrm{dom}(h) \cup \mathrm{img}(h)$. A location $\ell$ is *dangling* if $\ell \in \mathrm{img}(h) \setminus \mathrm{dom}(h)$. We write $\mathbf{S}$ for the set of all stacks and $\mathbf{H}$ for the set of all heaps.

*Two notions of disjoint union of heaps.* We write $h_1 + h_2$ for the union of disjoint heaps, i.e.,

$$h_1 + h_2 := \begin{cases} h_1 \cup h_2, & \text{if } \mathrm{dom}(h_1) \cap \mathrm{dom}(h_2) = \emptyset \\ \bot, & \text{otherwise.} \end{cases}$$

This standard notion of disjoint union is commonly used to assign semantics to the separating conjunction and magic wand. It requires that $h_1$ and $h_2$ are domain-disjoint, but does not impose any restrictions on the *images* of the heaps. In particular, the dangling pointers of $h_1$ may alias arbitrarily with the domain and image of $h_2$ and vice-versa.

Let $s$ be a stack. We write $h_1 \uplus^s h_2$ for the disjoint union of $h_1$ and $h_2$ that restricts aliasing of dangling pointers to the locations in stack $s$. This yields an infinite family of union operators: one for each stack. Formally,

$$h_1 \uplus^s h_2 := \begin{cases} h_1 + h_2, & \text{if } \mathsf{locs}(h_1) \cap \mathsf{locs}(h_2) \subseteq \mathrm{img}(s) \\ \bot, & \text{otherwise.} \end{cases}$$

Intuitively, $h_1 \uplus^s h_2$ is the (disjoint) union of heaps that share only locations that are in the image of the stack $s$. Note that if $h_1 \uplus^s h_2$ is defined then $h_1 + h_2$ is defined, but not vice-versa.

Just like the standard disjoint union $+$, the operator $\uplus^s$ gives rise to a separation algebra, i.e., a cancellative, commutative partial monoid [11]:

**Lemma 1.** *Let $s$ be a stack and let $u$ be the empty heap (i.e., $\mathrm{dom}(u) = \emptyset$). The triple $(\mathbf{H}, \uplus^s, u)$ is a separation algebra.*

*Weak- and strong-separation logic.* Both $+$ and $\uplus^s$ can be used to give a semantics to the separating conjunction and septraction. We denote the corresponding model relations $\models^{\mathsf{wk}}$ and $\models^{\mathsf{st}}$ and define them in Fig. 3. Where the two semantics agree, we simply write $\models$.

In both semantics, **emp** only holds for the empty heap, and $x = y$ holds for the empty heap when $x$ and $y$ are interpreted by the same location[4]. Points-to assertions $x \mapsto y$ are precise, i.e., only hold in singleton heaps. (It is, of course, possible to express intuitionistic points-to assertions by $x \mapsto y * \mathsf{t}$.) The list segment predicate $\mathtt{ls}(x, y)$ holds in possibly-empty lists of pointers from $s(x)$ to $s(y)$. The semantics of Boolean connectives are standard. The semantics of the separating conjunction, $*$, and septraction, $-\!\circledast$, differ based on the choice of $+$ vs. $\uplus^s$ for combining disjoint heaps. In the former case, denoted $\models^{\mathsf{wk}}$, we get the standard semantics of separation logic (cf. [40]). In the latter case, denoted $\models^{\mathsf{st}}$, we get a semantics that imposes stronger requirements on sub-heap composition: Sub-heaps may only overlap at locations that are stored in the stack.

[4] Usually $x = y$ is defined to hold for *all* heaps, not just the empty heap, when $x$ and $y$ are interpreted by the same location; however, this choice does not change the expressivity of the logic: the formula $(x = y) * \mathsf{t}$ expresses the standard semantics. Our choice is needed for the results on the positive fragment considered in Section 2.3

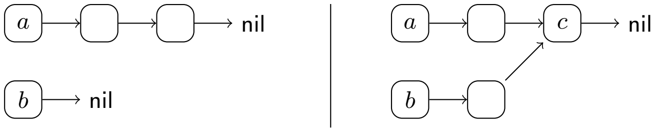

Fig. 4: Two models of $(\mathtt{ls}(a, \mathsf{nil}) * \mathtt{t}) \wedge (\mathtt{ls}(b, \mathsf{nil}) * \mathtt{t})$ for a stack with domain $a, b$ and a stack with domain $a, b, c$.

Because the semantics $\models^{\mathsf{st}}$ imposes stronger constraints, we will refer to the standard semantics $\models^{\mathsf{wk}}$ as the *weak* semantics of separation logic and to the semantics $\models^{\mathsf{st}}$ as the *strong* semantics of separation logic. Moreover, we use the terms *weak-separation logic* (WSL) and *strong-separation logic* (SSL) to distinguish between SL with the semantics $\models^{\mathsf{wk}}$ and $\models^{\mathsf{st}}$.

*Example 1.* Let $\varphi := a \neq b * (\mathtt{ls}(a, \mathsf{nil}) * \mathtt{t}) \wedge (\mathtt{ls}(b, \mathsf{nil}) * \mathtt{t})$. In Fig. 4, we show two models of $\varphi$. On the left, we assume that $a, b$ are the only program variables, whereas on the right, we assume that there is a third program variable $c$.

Note that the latter model, where the two lists overlap, is possible in SSL *only* because the lists come together at the location labeled by $c$. If we removed variable $c$ from the stack, the model would no longer satisfy $\varphi$ according to the strong semantics, because $\uplus^s$ would no longer allow splitting the heap at that location. Conversely, the model would still satisfy $\varphi$ with standard semantics.

This is a feature rather than a bug of SSL: By demanding that the user of SSL specify aliasing explicitly—for example by using the specification $\mathtt{ls}(a, c) * \mathtt{ls}(b, c) * \mathtt{ls}(c, \mathsf{nil}) \wedge c \neq \mathsf{nil}$—we rule out unintended aliasing effects. △

*Satisfiability and Semantic Consequence.* We define the notions of satisfiability and semantic consequence parameterized by a finite set of variables $\mathbf{x} \subseteq \mathbf{Var}$. For a formula $\varphi$ with $\mathsf{fvs}(\varphi) \subseteq \mathbf{x}$, we say that $\varphi$ is *satisfiable* w.r.t. $\mathbf{x}$ if there is a model $(s, h)$ with $\mathrm{dom}(s) = \mathbf{x}$ such that $(s, h) \models^{\mathsf{st}} \varphi$. We say that $\varphi$ *entails* $\psi$ w.r.t. $\mathbf{x}$, in signs $\varphi \models^{\mathsf{st}}_{\mathbf{x}} \psi$, if $(s, h) \models^{\mathsf{st}} \varphi$ then also $(s, h) \models^{\mathsf{st}} \psi$ for all models $(s, h)$ with $\mathrm{dom}(s) = \mathbf{x}$.

#### 2.3 Correspondence of Strong and Weak Semantics on Positive Formulas

We call an SL formula $\varphi$ *positive* if it does not contain $\neg$. Note that, in particular, this implies that $\varphi$ does *not* contain the magic wand $-\!\!*$ or the atom $\mathtt{t}$.

In models of positive formulas, all dangling locations are labeled by variables:

**Lemma 2.** *Let $\varphi$ be positive and $(s, h) \models^{\mathsf{wk}} \varphi$. Then,* $(\mathrm{img}(h) \setminus \mathrm{dom}(h)) \subseteq \mathrm{img}(s)$.

As every location shared by heaps $h_1$ $h_2$ in $h_1 + h_2$ is dangling either in $h_1$ or in $h_2$ (or both), the operations $+$ and $\uplus^s$ coincide on models of positive formulas:

**Lemma 3.** *Let $(s, h_1) \models^{\mathsf{wk}} \varphi_1$ and $(s, h_2) \models^{\mathsf{wk}} \varphi_2$ for positive formulas $\varphi_1, \varphi_2$. Then $h_1 + h_2 \neq \bot$ iff $h_1 \uplus^s h_2 \neq \bot$.*

Since the semantics coincide on atomic formulas by definition and on $*$ by Lemma 2, we can easily show that they coincide on all positive formulas:

**Lemma 4.** *Let* $\varphi$ *be a positive formula and let* $(s,h)$ *be a model. Then* $(s,h) \models^{\mathsf{wk}} \varphi$ *iff* $(s,h) \models^{\mathsf{st}} \varphi$.

By negating Lemma 4, we have that $\{(s,h) \mid (s,h) \models^{\mathsf{wk}} \varphi\} \neq \left\{(s,h) \mid (s,h) \models^{\mathsf{st}} \varphi\right\}$ implies that $\varphi$ contains negation, either explicitly or in the form of a magic wand or $\mathtt{t}$. In particular, Lemma 4 implies that the two semantics coincide on the popular *symbolic-heap fragment* of separation logic.[5]

We remark that formula $\varphi$ in Example 1 only employs $\mathtt{t}$ but not $\neg$, $-\!\!*$. Hence, even if only $\mathtt{t}$ would be added to the positive fragment, Lemma 4 would no longer hold. Likewise, Lemma 4 does not hold under intuitionistic semantics: as the intuitionistic semantics of a predicate $p$ is equivalent to $p * \mathtt{t}$ under classic semantics, if is sufficient to consider $\varphi := a \neq b * (\mathtt{ls}(a, \mathsf{nil}) \wedge (\mathtt{ls}(b, \mathsf{nil}))$.

### 3 Deciding the SSL Satisfiability Problem

The goal of this section is to develop a decision procedure for SSL:

**Theorem 1.** *Let* $\varphi \in \mathbf{SL}$ *and let* $\mathbf{x} \subseteq \mathbf{Var}$ *be a finite set of variables with* $\mathsf{fvs}(\varphi) \subseteq \mathbf{x}$. *It is decidable in* PSPACE *(in* $|\varphi|$ *and* $|\mathbf{x}|$*) whether there exists a model* $(s,h)$ *with* $\mathrm{dom}(s) = \mathbf{x}$ *and* $(s,h) \models^{\mathsf{st}} \varphi$.

Our approach is based on abstracting stack–heap models by *abstract memory states* (AMS), which have two key properties, which together imply Theorem 1:

**Refinement (Theorem 2).** If $(s_1, h_1)$ and $(s_2, h_2)$ abstract to the same AMS, then they satisfy the same formulas. That is, the AMS abstraction *refines* the satisfaction relation of SSL.

**Computability (Theorem 5, Lemmas 20 and 22).** For each formula $\varphi$, we can compute (in PSPACE) the set of all AMSs of all models of $\varphi$; then, $\varphi$ is satisfiable if this set is nonempty.

The AMS abstraction is motivated by the following insights.

1. The operator $\uplus^s$ induces a unique decomposition of the heap into at most $|s|$ minimal *chunks* of memory that cannot be further decomposed.
2. To decide whether $(s,h) \models^{\mathsf{st}} \varphi$ holds, it is sufficient to know for each chunk of $(s,h)$ a) which atomic formulas the chunk satisfies and b) which variables (if any) are allocated in the chunk.

[5] Strictly speaking, Lemma 4 implies this only for the symbolic-heap fragment of the separation logic studied in this paper, i.e., with the list predicate but no other data structures. The result can, however, be generalized to symbolic heaps with trees (see the dissertation of the first author [31]). Symbolic heaps of bounded treewidth as proposed in [21] are an interesting direction for future work.

We proceed as follows. In Sec 3.1, we make precise the notion of memory chunks. In Sec. 3.2, we define *abstract memory states* (AMS), an abstraction of models that retains for every chunk precisely the information from point (2) above. We will prove the *refinement theorem* in 3.3. We will show in Sections 3.4–3.6 that we can compute the AMS of the models of a given formula $\varphi$, which allows us to decide satisfiability and entailment problems for SSL. Finally, we prove the PSPACE-completeness result in Sec. 3.7.

#### 3.1 Memory Chunks

We will abstract a model $(s, h)$ by abstracting every *chunk* of $h$, which is a *minimal* nonempty sub-heap of $(s, h)$ that can be split off of $h$ according to the strong-separation semantics.

**Definition 1 (Sub-heap).** *Let $(s, h)$ be a model. We say that $h_1$ is a* sub-heap *of $h$, in signs $h_1 \sqsubseteq h$, if there is some heap $h_2$ such that $h = h_1 \uplus^s h_2$. We collect all sub-heaps in the set* $\mathsf{subHeaps}(s, h)$. △

The following proposition is an immediate consequence of the above definition:

**Proposition 1.** *Let $(s, h)$ be a model. Then, $(\mathsf{subHeaps}(s, h), \sqsubseteq, \sqcup, \sqcap, \neg)$ is a Boolean algebra with greatest element $h$ and smallest element $\emptyset$, where*

- $(s, h_1) \sqcup (s, h_2) := (s, h_1 \cup h_2)$,
- $(s, h_1) \sqcap (s, h_2) := (s, h_1 \cap h_2)$, *and*
- $\neg(s, h_1) := (s, h_1')$, *where $h_1' \in \mathsf{subHeaps}(s, h)$ is the unique sub-heap with $h = h_1 \uplus^s h_1'$.*

The fact that the sub-models form a Boolean algebra allows us to make the following definition[6]:

**Definition 2 (Chunk).** *Let $(s, h)$ be a model. A* chunk of $(s, h)$ *is an atom of the Boolean algebra $(\mathsf{subHeaps}(s, h), \sqsubseteq, \sqcup, \sqcap, \neg)$. We collect all chunks of $(s, h)$ in the set* $\mathsf{chunks}(s, h)$. △

Because every element of a Boolean algebra can be uniquely decomposed into atoms, we obtain that every heap can be fully decomposed into its chunks:

**Proposition 2.** *Let $(s, h)$ be a model and let $\mathsf{chunks}(s, h) = \{h_1, \ldots, h_n\}$ be its chunks. Then, $h = h_1 \uplus^s h_2 \uplus^s \cdots \uplus^s h_n$.*

*Example 2.* Let $s = \{x \mapsto 1, y \mapsto 3, u \mapsto 5, z \mapsto 3, w \mapsto 7, v \mapsto 9\}$ and $h = \{1 \mapsto 2, 2 \mapsto 3, 3 \mapsto 8, 4 \mapsto 6, 5 \mapsto 6, 6 \mapsto 3, 7 \mapsto 6, 9 \mapsto 9, 10 \mapsto 11, 11 \mapsto 10\}$. The model $(s, h)$ is illustrated in Fig. 5. This time, we include the identities of

[6] It is an interesting question for future work to relate the chunks considered in this paper to the atomic building blocks used in SL symbolic executions engines. Likewise, it would be interesting to build a symbolic execution engine based on the chunks resp. on the AMS abstraction proposed in this paper.

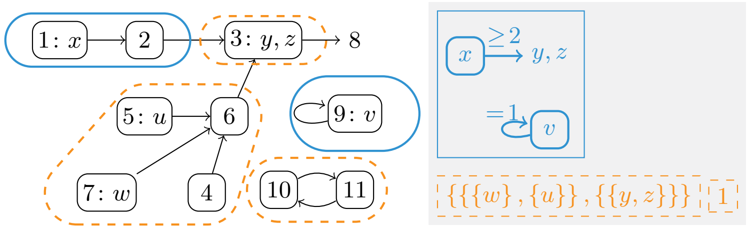

Fig. 5: Graphical representation of a model consisting of five chunks (left, see Ex. 2) and its induced AMS (right, see Ex. 5).

the locations in the graphical representation; e.g., $3\colon y, z$ represents location 3, $s(y) = 3$, $s(z) = 3$. The model consists of five chunks, $h_1 := \{1 \mapsto 2, 2 \mapsto 3\}$, $h_2 := \{9 \mapsto 9\}$, $h_3 := \{4 \mapsto 6, 5 \mapsto 6, 6 \mapsto 3, 7 \mapsto 6\}$, $h_4 := \{3 \mapsto 8\}$, and $h_5 := \{10 \mapsto 11, 11 \mapsto 10\}$. △

We distinguish two types of chunks: those that satisfy SSL atoms and those that don't.

**Definition 3 (Positive and Negative chunk).** *Let $h_c \subseteq h$ be a chunk of $(s, h)$. $h_c$ is a* positive chunk *if there exists an atomic formula $\tau$ such that $(s, h_c) \models^{\mathsf{st}} \tau$. Otherwise, $h_c$ is a* negative chunk*. We collect the respective chunks in* $\mathsf{chunks}^{+}(s, h)$ *and* $\mathsf{chunks}^{-}(s, h)$*.*

*Example 3.* Recall the chunks $h_1$ through $h_5$ from Ex. 2. $h_1$ and $h_2$ are positive chunks (blue in Fig. 5), $h_3$ to $h_5$ are negative chunks (orange). △

Negative chunks fall into three (not mutually-exclusive) categories:

**Garbage.** Chunks with locations that are inaccessible via stack variables.

**Unlabeled dangling pointers.** Chunks with an unlabeled sink, i.e., a dangling location that is not in $\mathrm{img}(s)$ and thus *cannot* be "made non-dangling" via composition using $\uplus^s$.

**Overlaid list segments.** Overlaid list segments that cannot be separated via $\uplus^s$ because they are joined at locations that are not in $\mathrm{img}(s)$.

*Example 4 (Negative chunks).* The chunk $h_3$ from Example 2 contains garbage, namely the location 4 that cannot be reached via stack variables, *and* two overlaid list segments (from 5 to 3 and 7 to 3). The chunk $h_4$ has an unlabeled dangling pointer. The chunk $h_5$ contains only garbage.

#### 3.2 Abstract Memory States

In *abstract memory states* (AMSs), we retain for every chunk enough information to (1) determine which atomic formulas the chunk satisfies, and (2) keep track of which variables are allocated within each chunk.

**Definition 4.** *A quadruple $\mathcal{A} = \langle V, E, \rho, \gamma \rangle$ is an* abstract memory state*, if*

1. *$V$ is a* partition *of some finite set of variables, i.e., $V = \{\mathbf{v}_1, \ldots, \mathbf{v}_n\}$ for some non-empty disjoint finite sets $\mathbf{v}_i \subseteq \mathbf{Var}$,*
2. *$E\colon V \rightharpoonup V \times \{=1, \geq 2\}$ is a partial function such that there is no $\mathbf{v} \in \mathrm{dom}(E)$ with $\mathsf{nil} \in \mathbf{v}$*[7]*,*
3. *$\rho$ consists of disjoint subsets of $V$ such that every $R \in \rho$ is disjoint from $\mathrm{dom}(E)$ and there is no $\mathbf{v} \in R$ with $\mathsf{nil} \in \mathbf{v}$,*
4. *$\gamma$ is a natural number, i.e., $\gamma \in \mathbb{N}$.*

*We call $V$ the* nodes*, $E$ the* edges*, $\rho$ the* negative-allocation constraint *and $\gamma$ the* garbage-chunk count *of $\mathcal{A}$. We call the AMS $\mathcal{A} = \langle V, E, \rho, \gamma\rangle$* garbage-free *if $\rho = \emptyset$ and $\gamma = \emptyset$.*

*We collect the set of all AMSs in* **AMS**. *The* size *of $\mathcal{A}$ is given by $|\mathcal{A}| := |V| + \gamma$. Finally, the* allocated variables *of an AMS are given by $\mathbf{alloc}(\mathcal{A}) := \mathrm{dom}(E) \cup \bigcup \rho$.* △

Every model induces an AMS, defined in terms of the following auxiliary definitions. The equivalence class of variable $x$ with regard to stack $s$ is $[x]^s_= := \{y \mid s(y) = s(x)\}$; the set of all equivalence classes of a given stack $s$ is $\mathsf{cls}_=(s) := \{[x]^s_= \mid x \in \mathrm{dom}(s)\}$. We now define the edges induced by a model $(s, h)$. For every equivalence class $[x]^s_= \in \mathsf{cls}_=(s)$, we set

$$\mathsf{edges}(s,h)([x]^s_=) := \begin{cases} \langle [y]^s_=, =1\rangle & \text{there are } y \in \mathrm{dom}(s) \text{ and } h_c \in \mathsf{chunks}^+(s,h) \\ & \quad \text{with } (s,h_c) \models^{\mathsf{st}} x \mapsto y \\ \langle [y]^s_=, \geq 2\rangle & \text{there are } y \in \mathrm{dom}(s) \text{ and } h_c \in \mathsf{chunks}^+(s,h) \\ & \quad \text{with } (s,h_c) \models^{\mathsf{st}} \mathtt{ls}(x,y) \wedge \neg x \mapsto y \\ \bot, & \text{otherwise.} \end{cases}$$

Finally, we denote the sets of variables allocated in negative chunks by

$$\mathsf{alloc}^-(s,h) := \{\{[x]^s_= \mid s(x) \in \mathrm{dom}(h_c)\} \mid h_c \in \mathsf{chunks}^-(s,h)\} \setminus \{\emptyset\},$$

where (equivalence classes of) variables that are allocated in the same negative chunk are grouped together in a set.

Now we are ready to define the *induced AMS* of a model.

**Definition 5.** *Let $(s,h)$ be a model. Let $V := \mathsf{cls}_=(s)$, $E := \mathsf{edges}(s,h)$, $\rho := \mathsf{alloc}^-(s,h)$ and $\gamma := \left|\mathsf{chunks}^-(s,h)\right| - \left|\mathsf{alloc}^-(s,h)\right|$.*

*Then, we say that $\mathsf{ams}(s,h) := \langle V, E, \rho, \gamma\rangle$ is the* induced AMS *of $(s,h)$.* △

*Example 5.* The induced AMS of the model $(s,h)$ from Ex. 2 is illustrated on the right-hand side of Fig. 5. The blue box depicts the graph $(V,E)$ induced by the positive chunks $h_1, h_2$; the negative chunks that allocate variables are abstracted to the set $\rho = \{\{\{w\},\{u\}\},\{\{y,z\}\}\}$ (note that the variables $w$ and $u$ are allocated in the chunk $h_3$ and the aliasing variables $y, z$ are allocated in $h_4$); and the garbage-chunk count is 1, because $h_5$ is the only negative chunk that does not allocate stack variables. △

[7] The edges of an AMS represent either a single pointer (case "$=1$") or a list segment of at least length (case "$\geq 2$").

Observe that the induced AMS is indeed an AMS:

**Proposition 3.** *Let $(s, h)$ be a model. Then* $\mathsf{ams}(s, h) \in \mathbf{AMS}$.

The reverse also holds: Every AMS is the induced AMS of at least one model; in fact, even of a model of linear size.

**Lemma 5 (Realizability of AMS).** *Let $\mathcal{A} = \langle V, E, \rho, \gamma \rangle$ be an AMS. There exists a model $(s, h)$ with* $\mathsf{ams}(s, h) = \mathcal{A}$ *whose size is linear in the size of $\mathcal{A}$.*

The following lemma demonstrates that we only need the $\rho$ and $\gamma$ components in order to be able to deal with negation and/or the magic wand:

**Lemma 6 (Models of Positive Formulas have Garbage-free Abstractions).** *Let $(s, h)$ be a model. If $(s, h) \models \varphi$ for a positive formula $\varphi$, then* $\mathsf{ams}(s, h)$ *is garbage-free.*

We abstract SL formulas by the set of AMS of their models:

**Definition 6.** *Let $s$ be a stack. The* **SL** abstraction *w.r.t. $s$, $\alpha_s \colon$* $\mathbf{SL} \to 2^{\mathbf{AMS}}$, *is given by*

$$\alpha_s(\varphi) := \{\mathsf{ams}(s, h) \mid h \in \mathbf{H}, \text{ and } (s, h) \overset{\mathsf{st}}{\models} \varphi\}. \qquad \triangle$$

Because AMSs do not retain any information about heap locations, just about aliasing, abstractions do not differ for stacks with the same equivalence classes:

**Lemma 7.** *Let $s, s'$ be stacks with* $\mathsf{cls}_=(s) = \mathsf{cls}_=(s')$. *Then $\alpha_s(\varphi) = \alpha_{s'}(\varphi)$ for all formulas $\varphi$.*

#### 3.3 The Refinement Theorem for SSL

The main goal of this section is to show the following *refinement theorem*:

**Theorem 2 (Refinement Theorem).** *Let $\varphi$ be a formula and let $(s, h_1)$, $(s, h_2)$ be models with* $\mathsf{ams}(s, h_1) = \mathsf{ams}(s, h_2)$. *Then $(s, h_1) \overset{\mathsf{st}}{\models} \varphi$ iff $(s, h_2) \overset{\mathsf{st}}{\models} \varphi$.*

We will prove this theorem step by step, characterizing the AMS abstraction of all atomic formulas and of the composed models before proving the refinement theorem. In the remainder of this section, we fix some model $(s, h)$.

*Abstract Memory States of Atomic Formulas* The empty-heap predicate **emp** is only satisfied by the empty heap, i.e., by a heap that consists of zero chunks:

**Lemma 8.** $(s, h) \models \mathbf{emp}$ *iff* $\mathsf{ams}(s, h) = \langle \mathsf{cls}_=(s), \emptyset, \emptyset, 0 \rangle$

**Lemma 9.** *1.* $(s, h) \models x = y$ *iff* $\mathsf{ams}(s, h) = \langle \mathsf{cls}_=(s), \emptyset, \emptyset, 0 \rangle$ *and* $[x]^s_= = [y]^s_=$.
*2.* $(s, h) \models x \neq y$ *iff* $\mathsf{ams}(s, h) = \langle \mathsf{cls}_=(s), \emptyset, \emptyset, 0 \rangle$ *and* $[x]^s_= \neq [y]^s_=$.

Models of points-to assertions consist of a single positive chunk of size 1:

**Lemma 10.** *Let* $E = \{[x]^s_= \mapsto \langle [y]^s_=, =1\rangle\}$. $(s,h) \models x \mapsto y$ *iff* $\mathsf{ams}(s,h) = \langle \mathsf{cls}_=(s), E, \emptyset, 0\rangle$.

Intuitively, the list segment $\mathtt{ls}(x,y)$ is satisfied by models $(s,h)$ that consist of zero or more positive chunks, corresponding to a (possibly empty) list from some equivalence class $[x]^s_=$ to $[y]^s_=$ via (zero or more) intermediate equivalence classes $[x_1]^s_=, \dots, [x_n]^s_=$. We will use this intuition to define abstract lists; this notion allows us to characterize the AMSs arising from abstracting lists.

**Definition 7.** *Let* $\mathcal{A} = \langle V, E, \rho, \gamma\rangle \in \mathbf{AMS}$*, $s$ be a stack and* $x, y \in \mathbf{Var}$. *We say $A$ is an* abstract list *w.r.t. $x$ and $y$, in signs* $\mathcal{A} \in \mathbf{AbstLists}(x,y)$*, iff*

1. $V = \mathsf{cls}_=(s)$,
2. $\rho = \emptyset$ *and* $\gamma = 0$, *and*
3. *we can pick nodes* $\mathbf{v}_1, \dots, \mathbf{v}_n \in V$ *and labels* $\iota_1, \dots, \iota_{n-1} \in \{=1, \geq 2\}$ *such that* $x \in \mathbf{v}_1$, $y \in \mathbf{v}_n$ *and* $E = \{\mathbf{v}_i \mapsto \langle \mathbf{v}_{i+1}, \iota_i\rangle \mid 1 \leq i < n\}$. △

**Lemma 11.** $(s,h) \models \mathtt{ls}(x,y)$ *iff* $\mathsf{ams}(s,h) \in \mathbf{AbstLists}(x,y)$.

*Abstract Memory States of Models composed by the Union Operator* Our next goal is to lift the union operator $\uplus^s$ to the abstract domain **AMS**. We will define an operator $\bullet$ with the following property:

$$\text{if } h_1 \uplus^s h_2 \neq \bot \text{ then } \mathsf{ams}(s, h_1 \uplus^s h_2) = \mathsf{ams}(s,h_1) \bullet (s,h_2).$$

AMS composition is a partial operation defined only on *compatible* AMS. Compatibility enforces (1) that the AMSs were obtained for equivalent stacks (i.e., for stacks $s, s'$ with $\mathsf{cls}_=(s) = \mathsf{cls}_=(s')$), and (2) that there is no double allocation.

**Definition 8 (Compatibility of AMSs).** *AMSs* $\mathcal{A}_1 = \langle V_1, E_1, \rho_1, \gamma_1\rangle$ *and* $\mathcal{A}_2 = \langle V_2, E_2, \rho_2, \gamma_2\rangle$ *are* compatible *iff (1)* $V_1 = V_2$ *and (2)* $\mathbf{alloc}(\mathcal{A}_1) \cap \mathbf{alloc}(\mathcal{A}_2) = \emptyset$.

Note that if $h_1 \uplus^s h_2$ is defined, then $\mathsf{ams}(s,h_1)$ and $\mathsf{ams}(s,h_2)$ are compatible. The converse is not true, because $\mathsf{ams}(s,h_1)$ and $\mathsf{ams}(s,h_2)$ may be compatible even if $\mathrm{dom}(h_1) \cap \mathrm{dom}(h_2) \neq \emptyset$.

AMS composition is defined in a point-wise manner on compatible AMSs and undefined otherwise.

**Definition 9 (AMS composition).** *Let* $\mathcal{A}_i = \langle V_i, E_i, \rho_i, \gamma_i\rangle$ *for* $i = 1, 2$ *be two AMS. The* composition *of* $\mathcal{A}_1, \mathcal{A}_2$ *is then given by*

$$\mathcal{A}_1 \bullet \mathcal{A}_2 := \begin{cases} \langle V_1, E_1 \cup E_2, \rho_1 \cup \rho_2, \gamma_1 + \gamma_2\rangle, & \text{if } \mathcal{A}_1, \mathcal{A}_2 \text{ compatible} \\ \bot, & \text{otherwise.} \end{cases}$$

**Lemma 12.** *Let $s$ be a stack and let $h_1, h_2$ be heaps. If* $h_1 \uplus^s h_2 \neq \bot$ *then* $\mathsf{ams}(s,h_1) \bullet \mathsf{ams}(s,h_2) \neq \bot$.

We next show that $\mathsf{ams}(s, h_1 \uplus^s h_2) = \mathsf{ams}(s, h_1) \bullet \mathsf{ams}(s, h_2)$ whenever $h_1 \uplus^s h_2$ is defined:

**Lemma 13 (Homomorphism of composition).** *Let $(s, h_1), (s, h_2)$ be models with $h_1 \uplus^s h_2 \neq \bot$. Then, $\mathsf{ams}(s, h_1 \uplus^s h_2) = \mathsf{ams}(s, h_1) \bullet \mathsf{ams}(s, h_2)$.*

To show the refinement theorem, we need one additional property of AMS composition. If an AMS $\mathcal{A}$ of a model $(s, h)$ can be decomposed into two smaller AMS $\mathcal{A} = \mathcal{A}_1 \bullet \mathcal{A}_2$, it is also possible to decompose the heap $h$ into smaller heaps $h_1, h_2$ with $\mathsf{ams}(s, h_i) = \mathcal{A}_i$:

**Lemma 14 (Decomposability of AMS).** *Let $\mathsf{ams}(s, h) = \mathcal{A}_1 \bullet \mathcal{A}_2$. There exist $h_1, h_2$ with $h = h_1 \uplus^s h_2$, $\mathsf{ams}(s, h_1) = \mathcal{A}_1$ and $\mathsf{ams}(s, h_2) = \mathcal{A}_2$.*

These results suffice to prove the Refinement Theorem stated at the beginning of this section (see the extended version [33] for a proof).

**Corollary 1.** *Let $(s, h)$ be a model and $\varphi$ be a formula. $(s, h) \models^{\mathsf{st}} \varphi$ iff $\mathsf{ams}(s, h) \in \alpha_s(\varphi)$.*

#### 3.4 Recursive Equations for Abstract Memory States

In this section, we derive recursive equations that reduce the set of AMS $\alpha_s(\varphi)$ for arbitrary compound formulas to the set of AMS of the constituent formulas of $\varphi$. In the next sections, we will show that we can actually evaluate these equations, thus obtaining an algorithm for computing the abstraction of arbitrary formulas.

**Lemma 15.** *$\alpha_s(\varphi_1 \wedge \varphi_2) = \alpha_s(\varphi_1) \cap \alpha_s(\varphi_2)$.*

**Lemma 16.** *$\alpha_s(\varphi_1 \vee \varphi_2) = \alpha_s(\varphi_1) \cup \alpha_s(\varphi_2)$.*

**Lemma 17.** *$\alpha_s(\neg\varphi_1) = \{\mathsf{ams}(s, h) \mid h \in \mathbf{H}\} \setminus \alpha_s(\varphi_1)$.*

*The Separating Conjunction* In Section 3.3, we defined the composition operation, $\bullet$, on pairs of AMS. We now lift this operation to sets of AMS $\mathbf{A}_1, \mathbf{A}_2$:

$$\mathbf{A}_1 \bullet \mathbf{A}_2 := \{\mathcal{A}_1 \bullet \mathcal{A}_2 \mid \mathcal{A}_1 \in \mathbf{A}_1, \mathcal{A}_2 \in \mathbf{A}_2, \mathcal{A}_1 \bullet \mathcal{A}_2 \neq \bot\}.$$

Lemma 13 implies that $\alpha_s$ is a homomorphism from formulas and $*$ to sets of AMS and $\bullet$:

**Lemma 18.** *For all $\varphi_1, \varphi_2$, $\alpha_s(\varphi_1 * \varphi_2) = \alpha_s(\varphi_1) \bullet \alpha_s(\varphi_2)$.*

*The septraction operator.* We next define an *abstract septraction operator* $-\!\bullet$ that relates to $\bullet$ in the same way that $-\!\circledast$ relates to $*$. For two sets of AMS $\mathbf{A}_1, \mathbf{A}_2$ we set:

$$\mathbf{A}_1 -\!\bullet \mathbf{A}_2 := \{\mathcal{A} \in \mathbf{AMS} \mid \text{ there exists } \mathcal{A}_1 \in \mathbf{A}_1 \text{ s.t. } \mathcal{A} \bullet \mathcal{A}_1 \in \mathbf{A}_2\}$$

Then, $\alpha_s$ is a homomorphism from formulas and $-\!\circledast$ to sets of AMS and $-\!\bullet$:

**Lemma 19.** *For all $\varphi_1, \varphi_2$, $\alpha_s(\varphi_1 -\!\circledast \varphi_2) = \alpha_s(\varphi_1) -\!\bullet \alpha_s(\varphi_2)$.*

#### 3.5 Refining the Refinement Theorem: Bounding Garbage

Even though we have now characterized the set $\alpha_s(\varphi)$ for every formula $\varphi$, we do not yet have a way to implement AMS computation: While $\alpha_s(\varphi)$ is finite if $\varphi$ is a spatial atom, the set is infinite in general; see the cases $\alpha_s(\neg\varphi)$ and $\alpha_s(\varphi_1 \mathbin{-\!\circledast} \varphi_2)$. However, we note that for a fixed stack $s$ only the garbage-chunk count $\gamma$ of an AMS $\langle V, E, \rho, \gamma\rangle \in \alpha_s(\varphi)$ can be of arbitrary size, while the size of the nodes $V$, the edges $E$ and the negative-allocation constraint $\rho$ is bounded by $|s|$. Fortunately, to decide the satisfiability of any fixed formula $\varphi$, it is *not* necessary to keep track of arbitrarily large garbage-chunk counts.

We introduce the *chunk size* $\lceil\varphi\rceil$ of a formula $\varphi$, which provides an upper bound on the number of chunks that may be necessary to satisfy and/or falsify the formula; $\lceil\varphi\rceil$ is defined as follows:

- $\lceil \mathbf{emp} \rceil = \lceil x \mapsto y \rceil = \lceil \mathtt{ls}(x,y) \rceil = \lceil x = y \rceil = \lceil x \neq y \rceil := 1$
- $\lceil \varphi * \psi \rceil := \lceil\varphi\rceil + \lceil\psi\rceil$
- $\lceil \varphi \mathbin{-\!\circledast} \psi \rceil := \lceil\psi\rceil$
- $\lceil \varphi \wedge \psi \rceil = \lceil \varphi \vee \psi \rceil := \max(\lceil\varphi\rceil, \lceil\psi\rceil)$
- $\lceil \neg\varphi \rceil := \lceil\varphi\rceil$.

Observe that $\lceil\varphi\rceil \leq |\varphi|$ for all $\varphi$. Intuitively, $\lceil\varphi\rceil - 1$ is an upper bound on the number of times the operation $\uplus^s$ needs to be applied when checking whether $(s,h) \overset{\mathsf{st}}{\models} \varphi$. For example, let $\psi := x \mapsto y * ((b \mapsto c) \mathbin{-\!\circledast} (\mathtt{ls}(a,c))$. Then $\lceil\psi\rceil = 2$, and to verify that $\psi$ holds in a model that consists of a pointer from $x$ to $y$ and a list segment from $a$ to $b$, it suffices to split this model $\lceil\varphi\rceil - 1 = 1$ many times using $\uplus^s$ (into the pointer and the list segment).

We generalize the refinement theorem, Theorem 2, to models whose AMS differ in their garbage-chunk count, provided both garbage-chunk counts exceed the chunk size of the formula:

**Theorem 3 (Refined Refinement Theorem).** *Let $\varphi$ be a formula with $\lceil\varphi\rceil = k$. Let $m \geq k$, $n \geq k$ and let $(s,h_1)$ and $(s,h_2)$ be models such that $\mathsf{ams}(s,h_1) = \langle V,E,\rho,m\rangle$, $\mathsf{ams}(s,h_2) = \langle V,E,\rho,n\rangle$. Then, $(s,h_1) \overset{\mathsf{st}}{\models} \varphi$ iff $(s,h_2) \overset{\mathsf{st}}{\models} \varphi$.*

This implies that $\varphi$ is satisfiable over stack $s$ iff $\varphi$ is satisfiable by a heap that contains at most $\lceil\varphi\rceil$ garbage chunks:

**Corollary 2.** *Let $\varphi$ be an formula with $\lceil\varphi\rceil = k$. Then $\varphi$ is satisfiable over stack $s$ iff there exists a heap $h$ such that (1) $\mathsf{ams}(s,h) = (V,E,\rho,\gamma)$ for some $\gamma \leq k$ and (2) $(s,h) \overset{\mathsf{st}}{\models} \varphi$.*

#### 3.6 Deciding SSL by AMS Computation

Due to Cor. 2, we can decide the SSL satisfiability problem by means of a function $\mathsf{abst}_s(\varphi)$ that computes the (finite) intersection of the (possibly infinite) set $\alpha_s(\varphi)$ and the (finite) set $\mathbf{AMS}_{k,s} := \{\langle V,E,\rho,\gamma\rangle \in \mathbf{AMS} \mid V = \mathsf{cls}_=(s) \text{ and } \gamma \leq k\}$ for $k = \lceil\varphi\rceil$. We define $\mathsf{abst}_s(\varphi)$ in Fig. 6. For atomic predicates we only need to consider garbage-chunk-count 0, whereas the cases $*$, $\mathbin{-\!\circledast}$, $\wedge$ and $\vee$ require *lifting* the bound on the garbage-chunk count from $m$ to $n \geq m$.

$$\begin{aligned}
\mathsf{abst}_s(\mathbf{emp}) &:= \{\langle \mathsf{cls}_=(s), \emptyset, \emptyset, 0\rangle\} \\
\mathsf{abst}_s(x = y) &:= \text{if } s(x) = s(y) \text{ then } \{\langle \mathsf{cls}_=(s), \emptyset, \emptyset, 0\rangle\} \text{ else } \emptyset \\
\mathsf{abst}_s(x \neq y) &:= \text{if } s(x) \neq s(y) \text{ then } \{\langle \mathsf{cls}_=(s), \emptyset, \emptyset, 0\rangle\} \text{ else } \emptyset \\
\mathsf{abst}_s(x \mapsto y) &:= \left\{\left\langle \mathsf{cls}_=(s), \left\{[x]^s_= \mapsto [y]^s_=\right\}, \emptyset, 0\right\rangle\right\} \\
\mathsf{abst}_s(\mathtt{ls}(x, y)) &:= \mathbf{AbstLists}(x, y) \cap \mathbf{AMS}_{0,s} \\
\mathsf{abst}_s(\varphi_1 * \varphi_2) &:= \mathbf{AMS}_{\lceil \varphi_1 * \varphi_2 \rceil, s} \cap \\
&\qquad (\mathsf{lift}_{\lceil \varphi_1 \rceil \nearrow \lceil \varphi_1 * \varphi_2 \rceil}(\mathsf{abst}_s(\varphi_1)) \bullet \mathsf{lift}_{\lceil \varphi_2 \rceil \nearrow \lceil \varphi_1 * \varphi_2 \rceil}(\mathsf{abst}_s(\varphi_2))) \\
\mathsf{abst}_s(\varphi_1 -\!\circledast \varphi_2) &:= \mathbf{AMS}_{\lceil \varphi_1 -\circledast \varphi_2 \rceil, s} \cap (\mathsf{abst}_s(\varphi_1) -\!\bullet \mathsf{lift}_{\lceil \varphi_2 \rceil \nearrow \lceil \varphi_1 \rceil + \lceil \varphi_2 \rceil}(\mathsf{abst}_s(\varphi_2))) \\
\mathsf{abst}_s(\varphi_1 \wedge \varphi_2) &:= \mathsf{lift}_{\lceil \varphi_1 \rceil \nearrow \lceil \varphi_1 \wedge \varphi_2 \rceil}(\mathsf{abst}_s(\varphi_1)) \cap \mathsf{lift}_{\lceil \varphi_2 \rceil \nearrow \lceil \varphi_1 \wedge \varphi_2 \rceil}(\mathsf{abst}_s(\varphi_2)) \\
\mathsf{abst}_s(\varphi_1 \vee \varphi_2) &:= \mathsf{lift}_{\lceil \varphi_1 \rceil \nearrow \lceil \varphi_1 \vee \varphi_2 \rceil}(\mathsf{abst}_s(\varphi_1)) \cup \mathsf{lift}_{\lceil \varphi_2 \rceil \nearrow \lceil \varphi_1 \vee \varphi_2 \rceil}(\mathsf{abst}_s(\varphi_2)) \\
\mathsf{abst}_s(\neg \varphi_1) &:= \mathbf{AMS}_{\lceil \varphi_1 \rceil, s} \setminus \mathsf{abst}_s(\varphi_1)
\end{aligned}$$

Fig. 6: Computing the abstract memory states of the models of $\varphi$ with stack $s$.

**Definition 10.** *Let $m, n \in \mathbb{N}$ with $m \leq n$ and let $\mathcal{A} = \langle V, E, \rho, \gamma\rangle \in \mathbf{AMS}$. The* bound-lifting *of $\mathcal{A}$ from $m$ to $n$ is*

$$\mathsf{lift}_{m \nearrow n}(\mathcal{A}) := \begin{cases} \{\mathcal{A}\} & \textit{if } \gamma < m \\ \{\langle V, E, \rho, k\rangle \mid m \leq k \leq n\} & \textit{if } \gamma = m. \end{cases}$$

*We generalize bound-lifting to sets of AMS:* $\mathsf{lift}_{m \nearrow n}(\mathbf{A}) := \bigcup_{\mathcal{A} \in \mathbf{A}} \mathsf{lift}_{m \nearrow n}(\mathcal{A})$. △

As a consequence of Theorem 3 bound-lifting is sound for all $n \geq \lceil \varphi \rceil$, i.e.,

$$\mathsf{lift}_{\lceil \varphi \rceil \nearrow n}(\alpha_s(\varphi) \cap \mathbf{AMS}_{\lceil \varphi \rceil}) = \alpha_s(\varphi) \cap \mathbf{AMS}_n.$$

By combining this observation with the lemmas characterizing $\alpha_s$, that is Lemmas 8,9,10, 11,15, 16,17, 18 and 19, we obtain the correctness of $\mathsf{abst}_s(\varphi)$:

**Theorem 4.** *Let $s$ be a stack and $\varphi$ be a formula. Then,* $\mathsf{abst}_s(\varphi) = \alpha_s(\varphi) \cap \mathbf{AMS}_{\lceil \varphi \rceil, s}$.

*Computability of* $\mathsf{abst}_s(\varphi)$*.* We note that the operators $\bullet, -\!\bullet, \cap, \cup$ and $\setminus$ are all computable as the sets that occur in the definition of $\mathsf{abst}_s(\varphi)$ are all finite. It remains to argue that we can compute the set of AMS for all atomic formulas. This is trivial for **emp**, (dis-)equalities, and points-to assertions. For the list-segment predicate, we note that the set $\mathsf{abst}_s(\mathtt{ls}(x, y)) = \mathbf{AbstLists}(x, y) \cap \mathbf{AMS}_{\lceil 0 \rceil, s}$ can be easily computed as there are only finitely many abstract lists w.r.t. the set of nodes $V = \mathsf{cls}_=(s)$. We obtain the following results:

**Corollary 3.** *Let $s$ be a (finite) stack. Then* $\mathsf{abst}_s(\varphi)$ *is computable for all formulas $\varphi$.*

**Theorem 5.** *Let $\varphi \in \mathbf{SL}$ and let $\mathbf{x} \subseteq \mathbf{Var}$ be a finite set of variables with* $\mathsf{fvs}(\varphi) \subseteq \mathbf{x}$*. It is decidable whether there exists a model $(s, h)$ with $\mathrm{dom}(s) = \mathbf{x}$ and $(s, h) \overset{\mathsf{st}}{\models} \varphi$.*

**Corollary 4.** *$\varphi \overset{\mathsf{st}}{\models}_{\mathbf{x}} \psi$ is decidable for all finite sets of variables $\mathbf{x} \subseteq \mathbf{Var}$ and $\varphi, \psi \in \mathbf{SL}$.*

$$\mathsf{qbf\_to\_sl}(F) := \mathbf{emp} \wedge \bigwedge_{\text{pairwise different QBF variables } x,y} x \neq y \wedge \mathsf{aux}(F)$$

$$\begin{aligned} \mathsf{aux}(x) &:= (x \mapsto \mathsf{nil}) * \mathsf{t} & \mathsf{aux}(\neg x) &:= \neg \mathsf{aux}(x) \\ \mathsf{aux}(F \wedge G) &:= \mathsf{aux}(F) \wedge \mathsf{aux}(G) & \mathsf{aux}(F \vee G) &:= \mathsf{aux}(F) \vee \mathsf{aux}(G) \\ \mathsf{aux}(\exists x.\, F) &:= (x \mapsto \mathsf{nil} \vee \mathbf{emp}) \mathbin{-\circledast} \mathsf{aux}(F) & \mathsf{aux}(\forall x.\, F) &:= (x \mapsto \mathsf{nil} \vee \mathbf{emp}) \mathbin{-\!\!*} \mathsf{aux}(F) \end{aligned}$$

Fig. 7: Translation $\mathsf{qbf\_to\_sl}(F)$ from closed QBF formula $F$ (in negation normal form) to a formula that is satisfiable iff $F$ is true.

#### 3.7 Complexity of the SSL Satisfiability Problem

It is easy to see that the algorithm $\mathsf{abst}_s(\varphi)$ runs in exponential time. We conclude this section with a proof that SSL satisfiability and entailment are actually PSpace-complete.

PSpace-*hardness.* An easy reduction from quantified Boolean formulas (QBF) shows that the SSL satisfiability problem is PSpace-hard. The reduction is presented in Fig. 7. We encode positive literals $x$ by $(x \mapsto \mathsf{nil}) * \mathsf{t}$ (the heap contains the pointer $x \mapsto \mathsf{nil}$) and negative literals by $\neg((x \mapsto \mathsf{nil}) * \mathsf{t})$ (the heap does not contain the pointer $x \mapsto \mathsf{nil}$). The magic wand is used to simulate universals (i.e., to enforce that we consider both the case $x \mapsto \mathsf{nil}$ and the case **emp**, setting $x$ both to true and to false). Analogously, septraction is used to simulate existentials. Similar reductions can be found (for standard SL) in [12].

**Lemma 20.** *The SSL satisfiability problem is* PSpace*-hard (even without the* `ls` *predicate).*

Note that this reduction simultaneously proves the PSpace-hardness of SSL model checking: If $F$ is a QBF formula over variables $x_1, \ldots, x_k$, then $\mathsf{qbf\_to\_sl}(F)$ is satisfiable iff $(\{x_i \mapsto l_i \mid 1 \leq i \leq n\}, \emptyset) \models^{\mathsf{st}} \mathsf{qbf\_to\_sl}(F)$ for some locations $\ell_i$ with $\ell_i \neq \ell_j$ for $i \neq j$.

PSpace-*membership.* For every stack $s$ and every bound on the garbage-chunk count of the AMS we consider, it is possible to encode every AMS by a string of polynomial length.

**Lemma 21.** *Let* $k \in \mathbb{N}$*, let* $s$ *be a stack and* $n := k + |s|$*. There exists an injective function* $\mathsf{encode}\colon \mathbf{AMS}_{k,s} \to \{0,1\}^*$ *such that*

$$|\mathsf{encode}(\mathcal{A})| \in \mathcal{O}(n \log(n)) \quad \textit{for all } \mathcal{A} \in \mathbf{AMS}_{k,s}.$$

An enumeration-based implementation of the algorithm in Fig. 6 (that has to keep in memory at most one AMS per subformula at any point in the computation) therefore runs in PSpace:

**Lemma 22.** *Let* $\varphi \in \mathbf{SL}$ *and let* $\mathbf{x} \subseteq \mathbf{Var}$ *be a finite set of variables with* $\mathsf{fvs}(\varphi) \subseteq \mathbf{x}$*. It is decidable in* PSpace *(in* $|\varphi|$ *and* $|\mathbf{x}|$*) whether there exists a model* $(s,h)$ *with* $\mathrm{dom}(s) = \mathbf{x}$ *and* $(s,h) \models^{\mathsf{st}} \varphi$*.*

The PSpace-completeness result, Theorem 1, follows by combining Lemmas 20 and 22.

$$\frac{}{\{x \mapsto z\}\, x.\mathsf{next} := y\, \{x \mapsto y\}} \qquad \frac{}{\{\mathbf{emp}\}\, \mathsf{malloc}(x)\, \{x \mapsto m\}}$$

$$\frac{}{\{x \mapsto z\}\, \mathsf{free}(x)\, \{\mathbf{emp}\}} \qquad \frac{}{\{\mathbf{emp}\}\, x := y\, \{x = y\}}$$

$$\frac{}{\{y \mapsto z\}\, x := y.\mathsf{next}\, \{y \mapsto z * x = z\}}\; x \text{ different from } y$$

$$\frac{}{\{\mathbf{emp}\}\, \mathsf{assume}(\varphi)\, \{\varphi\}}\; \varphi \text{ is } x = y \text{ or } x \neq y$$

Fig. 8: Local proof rules of program statements for forward symbolic execution.

$$\text{Frame rule}\; \frac{\{P\}\, c\, \{Q\}}{\{A * P\}\, c\, \{A[\mathbf{x}'/\mathbf{x}] * Q\}}\; \mathbf{x} = \mathsf{modifiedVars}(c),\ \mathbf{x}' \text{ fresh}$$

$$\text{Materialization}\; \frac{\{P\}\, c\, \{Q\}}{\{P\}\, c\, \{x \mapsto z * ((x \mapsto z) -\!\circledast Q)\}}\; Q \models^{\mathsf{st}} \neg((x \mapsto \mathsf{nil}) -\!\circledast \mathsf{t}),\ z \text{ fresh}$$

Fig. 9: The frame and the materialization rule for forward symbolic execution.

### 4 Program Verification with Strong-Separation Logic

Our main practical motivation behind SSL is to obtain a decidable logic that can be used for fully automatically discharging verification conditions in a Hoare-style verification proof. Discharging VCs can be automated by calculi that symbolically execute pre-conditions forward resp. post-conditions backward, and then invoking an entailment checker. Symbolic execution calculi typically either introduce first-order quantifiers or fresh variables in order to deal with updates to the program variables. We leave the extension of SSL to support for quantifiers for future work and in this paper develop a forward symbolic execution calculus based on fresh variables.

We target the usual Hoare-style setting where a verification engineer annotates the pre- and post-condition of a function and provides loop invariants. We exemplify two annotated functions in Fig. 10; the left function reverses a list and the right function copies a list. In addition to the program variables, our annotations may contain logical variables (also known as ghost variables); for example, the annotations of list reverse only contain program variables, while the annotations of list copy also contain the logical variable $u$ (which is assumed to be equal to $x$ in the pre-condition)[8].

*Forward Symbolic Execution Rules.* We state local proof rules for a simple heap-manipulating programming language in Fig. 8. We remark that we do not include a rule for the statement $\mathsf{x} := \mathsf{x.next}$ for ease of exposition; however, this is w.l.o.g. because $\mathsf{x} := \mathsf{x.next}$ can be simulated by the statements $\mathsf{y} := \mathsf{x.next}; \mathsf{x} := \mathsf{y}$ at the expense of introducing an additional program variable $y$. Our only non-standard choice is the modelling of the $\mathsf{malloc}$ statement: we assume a special program variable $m$, which is never referenced by any program statement and only used

[8] $m$ is a special program variable introduced for modelling $\mathsf{malloc}$.

in the modelling; the malloc statement updates the value of the variable $m$ to the target of the newly allocated memory cell; this modelling justifies the proof rule for malloc stated in Fig. 8. For a small-step operational semantics of our program statements we refer the reader to the extended version [33]. The rules for the program statements in Fig. 8 are local in the sense that they only deal with a single pointer or the empty heap. The rules in Fig. 9 are the main rules of our forward symbolic execution calculus. The frame rule is essential for lifting the local proof rules to larger heaps. The materialization rule ensures that the frame rule can be applied whenever the pre-condition of a local proof rule can be met. We now give more details. For a sequence of program statements $\mathbf{c} = c_1 \cdots c_k$ and a pre-condition $P_{start}$ the symbolic execution calculus derives triples $\{P_{start}\}\, c_1 \cdots c_i\, \{Q_i\}$ for all $1 \leq i \leq k$. In order to proceed from $i$ to $i+1$, either 1) only the frame rule is applied or 2) the materialization rule is applied first followed by an application of the frame rule. The frame rule can be applied if the formula $Q_i$ has the shape $Q_i = A * P$, where $A$ is suitably chosen and $P$ is the pre-condition of the local proof rule for statement $c_i$. Then, $Q_{i+1}$ is given by $Q_{i+1} = A[\mathbf{x}'/\mathbf{x}] * Q$, where $\mathbf{x} = \mathsf{modifiedVars}(c)$, $\mathbf{x}'$ are fresh copies of the variables $\mathbf{x}$ and $Q$ is the right hand side of the local proof rule for statement $c_i$, i.e., we have $\{P\}\, c_i\, \{Q\}$. Note that the frame rule requires substituting the modified program variables with fresh copies: We set $\mathsf{modifiedVars}(c) := \{x, m\}$ for $c = \mathsf{malloc}(x)$, $\mathsf{modifiedVars}(c) := \{x\}$ for $c = \mathsf{x := y.next}$ and $c = \mathsf{x := y}$, and $\mathsf{modifiedVars}(c) := \emptyset$, otherwise. The materialization rule may be applied in order to ensure that $Q_i$ has the shape $Q_i = A * P$. This is not needed in case $P = \mathbf{emp}$ but may be necessary for $P = x \mapsto y$. We note that $Q_i$ guarantees that a pointer $x$ is allocated iff $Q_i \models^{\mathsf{st}} \neg((x \mapsto \mathsf{nil}) \circledast \mathsf{t})$. Under this condition, the rule allows introducing a name $z$ for the target of the pointer $x$. We remark that while backward-symbolic execution calculi typically employ the magic wand, our forward calculus makes use of the dual septraction operator; we were able to design a general rule that guarantees a predicate of shape $Q_i = A * P$ without the need of coming up with dedicated rules for, e.g., unfolding list predicates.

*Applying the forward symbolic execution calculus for verification.* We now explain how the proof rules presented in Fig. 8 and 9 can be used for program verification. Our goal is to verify that the pre-condition $P$ of a loop-free piece of code $c$ (in our case, a sequence of program statements) implies the post-condition $Q$. For this, we apply the symbolic execution calculus and derive a triple $\{P\}\, c\, \{Q'\}$. It then remains to verify that the final state of the symbolic execution $Q'$ implies the post-condition $Q$. Here, we face the difficulty that the symbolic execution introduces additional variables: Let us assume that all annotations are over a set of variables $\mathbf{x}$, which includes the program variables and the logical variables. Further assume that the symbolic execution $\{P\}\, c\, \{Q'\}$ introduced the fresh variables $\mathbf{y}$. With the results of Section 3 we can then verify the entailment $Q' \models^{\mathsf{st}}_{\mathbf{x} \cup \mathbf{y}} Q$. However, we need to guarantee that all models $(s, h)$ of $Q$ with $\mathrm{dom}(s) = \mathbf{x} \cup \mathbf{y}$ are also models when we restrict $\mathrm{dom}(s)$ to $\mathbf{x}$ (note that we can think of the variables $\mathbf{y}$ as implicitly existentially quantified). In order to deal with this issue, we require annotations to be robust:

```
{ls(x, nil)} % list reverse
    a := nil;
    while(x ≠ nil)
    {ls(x, nil) * ls(a, nil)}
    {   b := x.next;
        x.next := a;
        a := x;
        w := b;    }
    x := w;
{ls(x, nil)}
```

```
{ls(x, nil) * u = x} % list copy
    malloc(s);
    r := s;
    while(x ≠ nil)
    {ls(u, x) * ls(x, nil) * ls(r, s) * s ↦ m}
    {   malloc(t);
        % t.data := x.data; not modelled
        s.next := t;
        s := t;
        y := x.next;
        x := y;    }
    s.next := nil;
{ls(u, nil) * ls(r, nil)}
```

Fig. 10: List reverse (left) and list copy (right) annotated pre- and post-condition and loop invariants.

**Definition 11 (Robust Formula).** *We call a formula* $\varphi \in \mathbf{SL}$ robust*, if for all models* $(s_1, h)$ *and* $(s_2, h)$ *with* $\mathsf{fvs}(\varphi) \subseteq \mathrm{dom}(s_1)$ *and* $\mathsf{fvs}(\varphi) \subseteq \mathrm{dom}(s_2)$ *and* $s_1(x) = s_2(x)$ *for all* $x \in \mathsf{fvs}(\varphi)$*, we have that* $(s_1, h) \overset{\mathsf{st}}{\models} \varphi$ *iff* $(s_2, h) \overset{\mathsf{st}}{\models} \varphi$*.*

**Lemma 23.** *Let* $\varphi \in \mathbf{SL}$ *be a positive formula. Then,* $\varphi$ *is robust.*

Lemma 4 states that all formulas from the positive fragment are robust. In particular, the annotations in Fig. 10 are robust. As an example for a non-robust formula consider $\varphi$ in Example 1. We note that Lemma 4 does not cover all robust formulas, e.g., $\mathsf{t}$ is robust. We leave the identification of further robust formulas for future work.

We now state the soundness of our symbolic execution calculus:

**Lemma 24 (Soundness of Forward Symbolic Execution).** *Let* $\mathbf{c}$ *be a sequence of program statements, let* $P$ *be a robust formula, let* $\{P\}\,\mathbf{c}\,\{Q\}$ *be the triple obtained from symbolic execution, and let* $V$ *be the fresh variables introduced during symbolic execution. Then,* $Q$ *is robust and for all stack-heap pairs* $(s, h), (s', h')$ *such that* $(s, h) \overset{\mathsf{st}}{\models} P$ *and* $(s', h')$ *can be obtained from* $(s, h)$ *by executing* $\mathbf{c}$*, there is a stack* $s''$ *with* $s' \subseteq s''$*,* $V \subseteq \mathrm{dom}(s'')$ *and* $(s'', h') \overset{\mathsf{st}}{\models} Q$*.*

*Automation.* We note that the presented approach can fully-automatically verify that the pre-condition of a loop-free piece of code guarantees its post-condition: For every program statement, we apply its local proof rule using the frame rule (and in addition the materialization rule in case the existence of a pointer target must be guaranteed). We then discharge the entailment query using our decision procedure from Section 3. We now illustrate this approach on the programs from Fig. 10. For both programs we verify that the loop invariant is inductive (in both cases the loop-invariant $P$ is propagated forward through the loop body; it is then checked that the obtained formula $Q$ again implies the loop invariant $P$; for verifying the implication we apply our decision procedure from Corollary 4):

*Example 6.* Verifying the loop invariant of list reverse:

$\{\mathtt{ls}(x, \mathsf{nil}) * \mathtt{ls}(a, \mathsf{nil})\}\ (=: P)$

$\mathtt{assume}(\mathtt{x} \neq \mathsf{nil})$

$\{\mathtt{ls}(x, \mathsf{nil}) * \mathtt{ls}(a, \mathsf{nil}) * x \neq \mathsf{nil}\}$

`# materialization`

$\{x \mapsto z \,-\!\circledast (\mathtt{ls}(x, \mathsf{nil}) * \mathtt{ls}(a, \mathsf{nil}) * x \neq \mathsf{nil}) * x \mapsto z\}$

$\mathtt{b} := \mathtt{x.next}$

$\{x \mapsto z \,-\!\circledast (\mathtt{ls}(x, \mathsf{nil}) * \mathtt{ls}(a, \mathsf{nil}) * x \neq \mathsf{nil}) * x \mapsto z * b = z\}$

$\mathtt{x.next} := \mathtt{a}$

$\{x \mapsto z \,-\!\circledast (\mathtt{ls}(x, \mathsf{nil}) * \mathtt{ls}(a, \mathsf{nil}) * x \neq \mathsf{nil}) * x \mapsto a * b = z\}$

$\mathtt{a} := \mathtt{x}$

$\{x \mapsto z \,-\!\circledast (\mathtt{ls}(x, \mathsf{nil}) * \mathtt{ls}(a', \mathsf{nil}) * x \neq \mathsf{nil}) * x \mapsto a' * b = z * a = x\}$

$\mathtt{x} := \mathtt{b}$

$\{x' \mapsto z \,-\!\circledast (\mathtt{ls}(x', \mathsf{nil}) * \mathtt{ls}(a', \mathsf{nil}) * x' \neq \mathsf{nil}) * x' \mapsto a' * b = z *$
$a = x' * x = b\}(=: Q)$

$\{\mathtt{ls}(x, \mathsf{nil}) * \mathtt{ls}(a, \mathsf{nil})\}\ (=: P)$

*Example 7.* Verifying the loop invariant of list copy:

$\{\mathtt{ls}(u, x) * \mathtt{ls}(x, \mathsf{nil}) * \mathtt{ls}(r, s) * s \mapsto m\}\ (=: P)$

$\mathtt{assume}(\mathtt{x} \neq \mathsf{nil})$

$\{\mathtt{ls}(u, x) * \mathtt{ls}(x, \mathsf{nil}) * \mathtt{ls}(r, s) * s \mapsto m * x \neq \mathsf{nil}\}$

$\mathtt{malloc(t)}$

$\{\mathtt{ls}(u, x) * \mathtt{ls}(x, \mathsf{nil}) * \mathtt{ls}(r, s) * s \mapsto m' * x \neq \mathsf{nil} * t \mapsto m\}$

$\mathtt{s.next} := \mathtt{t}$

$\{\mathtt{ls}(u, x) * \mathtt{ls}(x, \mathsf{nil}) * \mathtt{ls}(r, s) * s \mapsto t * x \neq \mathsf{nil} * t \mapsto m\}$

$\mathtt{s} := \mathtt{t}$

$\{\mathtt{ls}(u, x) * \mathtt{ls}(x, \mathsf{nil}) * \mathtt{ls}(r, s') * s' \mapsto t * x \neq \mathsf{nil} * t \mapsto m * s = t\}$

`# materialization`

$\{x \mapsto z \,-\!\circledast (\mathtt{ls}(u, x) * \mathtt{ls}(x, \mathsf{nil}) * \mathtt{ls}(r, s') * s' \mapsto t * x \neq \mathsf{nil} * t \mapsto m * s = t) *$
$x \mapsto z\}$

$\mathtt{y} := \mathtt{x.next}$

$\{x \mapsto z \,-\!\circledast (\mathtt{ls}(u, x) * \mathtt{ls}(x, \mathsf{nil}) * \mathtt{ls}(r, s') * s' \mapsto t * x \neq \mathsf{nil} * t \mapsto m * s = t) *$
$x \mapsto z * y = z\}$

$\mathtt{x} := \mathtt{y}$

$\{x' \mapsto z \,-\!\circledast (\mathtt{ls}(u, x') * \mathtt{ls}(x', \mathsf{nil}) * \mathtt{ls}(r, s') * s' \mapsto t *$
$x' \neq \mathsf{nil} * t \mapsto m * s = t) * x' \mapsto z * y = z * x = y\}(=: Q)$

$\{\mathtt{ls}(u, x) * \mathtt{ls}(x, \mathsf{nil}) * \mathtt{ls}(r, s) * s \mapsto m\}\ (=: P)$

While our decision procedure can automatically discharge the entailments in both of the above examples, we give a short direct argument for the benefit of the reader for the entailment check of Example 6 (a direct argument could similarly be worked out for Example 7): We note that $Q'$ simplifies to $Q'' = \{a \mapsto x \text{-}\circledast(\texttt{ls}(a, \mathsf{nil}) * \texttt{ls}(a', \mathsf{nil})) * a \mapsto a'\}$. Every model $(s, h)$ of $Q''$ must consist of a pointer $a \mapsto a'$, a list segment $\texttt{ls}(a', \mathsf{nil})$ and a heap $h'$ to which the pointer $a \mapsto x$ can be added in order to obtain the list segment $\texttt{ls}(a, \mathsf{nil})$; by looking at the semantics of the list segment predicate we see that $h'$ in fact must be the list segment $\texttt{ls}(x, \mathsf{nil})$. Further, the pointer $a \mapsto a'$ can be composed with the list segment $\texttt{ls}(a', \mathsf{nil})$ in order to obtain $\texttt{ls}(a, \mathsf{nil})$.

### 5 Normal Forms and the Abduction Problem

In this section, we discuss how every AMS can be expressed by a formula, which in turn makes it possible to compute a normal form for every formula. We then discuss how the normal form transformation has applications to the abduction problem.

*Normal Form.* We lift the abstraction functions from stacks to sets of variables: Let $\mathbf{x} \subseteq \mathbf{Var}$ be a finite set of variables and $\varphi \in \mathbf{SL}$ be a formula with $\mathsf{fvs}(\varphi) \subseteq \mathbf{x}$. We set $\alpha_{\mathbf{x}}(\varphi) := \{\alpha_{\mathbf{s}}(\varphi) \mid \mathrm{dom}(s) = \mathbf{x}\}$ and $\mathsf{abst}_{\mathbf{x}}(\varphi) := \alpha_{\mathbf{x}}(\varphi) \cap \mathbf{AMS}_{\lceil \varphi \rceil, \mathbf{x}}$, where $\mathbf{AMS}_{k,\mathbf{x}} := \{\langle V, E, \rho, \gamma \rangle \in \mathbf{AMS} \mid \bigcup V = \mathbf{x} \text{ and } \gamma \leq k\}$. (We note that $\alpha_{\mathbf{x}}(\varphi)$ is computable by the same argument as in the proof of Theorem 5.)

**Definition 12 (Normal Form).** *Let* $\mathsf{NF}_{\mathbf{x}}(\varphi) := \bigvee_{\mathcal{A} \in \alpha_{\mathbf{x}}(\varphi)} \mathsf{AMS2SL}^{\lceil \varphi \rceil}(\mathcal{A})$ *the* normal form *of* $\varphi$*, where* $\mathsf{AMS2SL}^m(\mathcal{A})$ *is defined as in Fig. 11.* △

The definition of $\mathsf{AMS2SL}^m(\mathcal{A})$ represents a straightforward encoding of the AMS $\mathcal{A}$: *aliasing* encodes the aliasing between the stack variables as implied by $V$; *graph* encodes the points-to assertions and lists of length at least two corresponding to the edges $E$; *negalloc* encodes that the negative chunks $R \in \rho$ precisely allocate the variables $\mathbf{v} \in R$; *garbage* ensures that there are either exactly $\gamma$ additional non-empty memory chunks that do not allocate any stack variable (if $\gamma < m$) or at least $\gamma$ such chunks (if $\gamma = m$); *negalloc* and *garbage* use the formula *negchunk* which precisely encodes the definition of a negative chunk. We have the following result about normal forms:

**Theorem 6.** $\mathsf{NF}_{\mathbf{x}}(\varphi) \models^{\mathsf{st}}_{\mathbf{x}} \varphi$ *and* $\varphi \models^{\mathsf{st}}_{\mathbf{x}} \mathsf{NF}_{\mathbf{x}}(\varphi)$*.*

*The abduction problem.* We consider the following relaxation of the entailment problem: The *abduction problem* is to replace the question mark in the entailment $\varphi * [?] \models^{\mathsf{st}}_{\mathbf{x}} \psi$ by a formula such that the entailment becomes true. This problem

$$\begin{aligned}
\mathsf{AMS2SL}^m(\mathcal{A}) :=& \mathsf{aliasing}(\mathcal{A}) * \mathsf{graph}(\mathcal{A}) * \mathsf{negalloc}(\mathcal{A}) * \mathsf{garbage}^m(\mathcal{A}) \\
\mathsf{aliasing}(\mathcal{A}) :=& \left(\mathop{\Large *}_{\mathbf{v}\in V, x,y\in\mathbf{v}} x = y\right) * \left(\mathop{\Large *}_{\mathbf{v},\mathbf{w}\in V, \mathbf{v}\neq\mathbf{w}} \max(\mathbf{v}) \neq \max(\mathbf{w})\right) \\
\mathsf{graph}(\mathcal{A}) :=& \left(\mathop{\Large *}_{E(\mathbf{v})=\langle\mathbf{v}',=1\rangle} \max(\mathbf{v}) \mapsto \max(\mathbf{v}')\right) * \\
& \left(\mathop{\Large *}_{E(\mathbf{v})=\langle\mathbf{v}',\geq 2\rangle} \mathtt{ls}_{\geq 2}(\max(\mathbf{v}), \max(\mathbf{v}'))\right) \\
\mathsf{negalloc}(\mathcal{A}) :=& \mathop{\Large *}_{R\in\rho} \mathsf{negchunk}(\mathcal{A}) \wedge \bigwedge_{\mathbf{v}\in R} \mathsf{alloc}(\max(\mathbf{v})) \wedge \bigwedge_{\mathbf{v}\in V\setminus R} \neg\mathsf{alloc}(\max(\mathbf{v})) \\
\mathsf{garbage}^m(\mathcal{A}) :=& \begin{cases} \mathsf{garbage}(\mathcal{A},\gamma) & \text{if } \gamma < m \\ \mathsf{garbage}(\mathcal{A}, m\text{-}1) * \neg\mathbf{emp} \bigwedge_{\mathbf{v}\in V} \neg\mathsf{alloc}(\max(\mathbf{v})) & \text{otherwise} \end{cases} \\
\mathsf{garbage}(\mathcal{A},k) :=& \begin{cases} \mathbf{emp} & \text{if } k = 0 \\ \mathsf{garbage}(\mathcal{A}, k\text{-}1) * \mathsf{negchunk}(\mathcal{A}) \wedge \bigwedge_{\mathbf{v}\in V} \neg\mathsf{alloc}(\max(\mathbf{v})) & \text{otherwise} \end{cases} \\
\mathsf{negchunk}(\mathcal{A}) :=& \neg\mathbf{emp} \wedge \neg(\neg\mathbf{emp} * \neg\mathbf{emp}) \wedge \\
& \bigwedge_{\mathbf{v},\mathbf{w}\in V, \varphi\in\{\max(\mathbf{v})\mapsto\max(\mathbf{w}), \mathtt{ls}(\max(\mathbf{v}),\max(\mathbf{w}))\}} \neg\varphi \\
\mathsf{alloc}(x) :=& \neg((x \mapsto \mathsf{nil}) \mathrel{-\!\!\circledast} \mathsf{t}) \\
\mathtt{ls}_{\geq 2}(x,y) :=& \mathtt{ls}(x,y) \wedge \neg(x \mapsto y)
\end{aligned}$$

Fig. 11: The induced formula $\mathsf{AMS2SL}^m(\mathcal{A})$ of AMS $\mathcal{A} = \langle V, E, \rho, \gamma\rangle$ with $\gamma \leq m$.

is central for obtaining a scalable program analyzer as discussed in [10] [9]. The abduction problem does in general not have a unique solution. Following [10], we thus consider optimization versions of the abduction problem, looking for *logically weakest* and *spatially minimal* solutions:

**Definition 13.** *Let $\varphi, \psi \in$ **SL** and $\mathbf{x} \subseteq$ **Var** be a finite set of variables. A formula $\zeta$ is the* weakest *solution to the abduction problem $\varphi * [?] \models^{\mathsf{st}}_{\mathbf{x}} \psi$ if it holds for all abduction solutions $\zeta'$ that $\zeta' \models^{\mathsf{st}}_{\mathbf{x}} \zeta$. An abduction solution is $\zeta$* minimal, *if there is no abduction solution $\zeta'$ with $\zeta \models^{\mathsf{st}}_{\mathbf{x}} \zeta' * (\neg\mathbf{emp})$.*

**Lemma 25.** *Let $\varphi, \psi$ be formulas and let $\mathbf{x} \subseteq$ **Var** be a finite set of variables. Then, 1) the weakest solution to the abduction problem $\varphi * [?] \models^{\mathsf{st}}_{\mathbf{x}} \psi$ is given by the formula $\varphi \mathrel{-\!\!*} \psi$, and the 2) weakest minimal solution is given by the formula $\varphi \mathrel{-\!\!*} \psi \wedge \neg((\varphi \mathrel{-\!\!*} \psi) * \neg\mathbf{emp})$.*

[9] While the program analyzer proposed in [10] employs *bi-abductive* reasoning, the bi-abduction procedure in fact proceeds in two separate abduction and frame-inference steps, where the main technical challenge is the abduction step, as frame inference can be incorporated into entailment checking. We believe that the situation for SSL is similar, i.e., solving abduction is the key to implementing a bi-abductive prover for SSL; hence, our focus on the abduction problem.

We now explain how the normal form has applications to the abduction problem. According to Lemma 25, the best solutions to the abduction problem are given by the formulas $\zeta := \varphi \mathrel{-\!\!*} \psi$ and $\zeta' := \varphi \mathrel{-\!\!*} \psi \wedge \neg((\varphi \mathrel{-\!\!*} \psi) * \neg\mathbf{emp})$. While it is great that the existence of these solutions is guaranteed, we a-priori do not have a means to *compute* an explicit representation of these solutions nor to further analyze their structure. However, the normal form operator allows us to obtain the explicit representations $\mathsf{NF}_{\mathbf{x}}(\zeta)$ and $\mathsf{NF}_{\mathbf{x}}(\zeta')$. We believe that using these explicit representations in a program analyzer or studying their properties is an interesting topic for further research. Here, we establish one concrete result on solutions to the abduction problem based on normal forms:

*We can compute the weakest resp. the weakest minimal solution to the abduction problem from the positive fragment.* Observe that among the sub-formulas of $\mathsf{aliasing}$ and $\mathsf{graph}$, only the formula $\mathtt{ls}_{\geq 2}$ is negative. To be able to use $\mathtt{ls}_{\geq 2}(x, y)$ in a positive formula, we first need to add a new spatial atom $\mathtt{ls}_{\geq 2}(x, y)$ to SSL with the following semantics: $\mathtt{ls}_{\geq 2}(x, y)$ holds in a model iff the model is a list segment of length at least 2 from $x$ to $y$. (The whole development in Sections 2 and 3 can be extended by this predicate.) We can then simplify the formula $\mathsf{graph}(\mathcal{A})$ in $\mathsf{AMS2SL}^m(\mathcal{A})$ by directly translating edges $E(\mathbf{v}) = \langle \mathbf{v}', \geq 2\rangle$ to the atom $\mathtt{ls}_{\geq 2}(\max(\mathbf{v}), \max(\mathbf{v}'))$. Then, $\bigvee_{\langle V,E,\rho,\gamma\rangle \in \alpha_{\mathbf{x}}(\zeta) \text{ with } \rho=\emptyset, \gamma=0} \mathsf{AMS2SL}^{\lceil \varphi \rceil}(\mathcal{A})$ for $\zeta = \varphi \mathrel{-\!\!*} \psi$ or $\zeta = \varphi \mathrel{-\!\!*} \psi \wedge \neg((\varphi \mathrel{-\!\!*} \psi) * \neg\mathbf{emp})$ is the weakest resp. the weakest minimal solution to the abduction problem from the positive fragment.

### 6 Conclusion

We have shown that the satisfiability problem for "strong" separation logic with lists is in the same complexity class as the satisfiability problem for standard "weak" separation logic without any data structures: PSPACE-complete. This is in stark contrast to the undecidability result for standard (weak) SL semantics, as shown in [16].

We have demonstrated the potential of SSL for program verification: 1) We have provided symbolic execution rules that, in conjunction with our result on the decidability of entailment, can be used for fully-automatically discharging verification conditions. 2) We have discussed how to compute explicit representations to optimal solutions of the abduction problem. This constitutes the first work that addresses the abduction problem for a separation logic closed under Boolean operators and the magic wand.

We consider our results just the first steps in examining strong-separation logic, motivated by the desire to circumvent the undecidability result of [16]. Future work is concerned with the practical evaluation of our decision procedures, with extending the symbolic execution calculus to a full Hoare logic as well as extending the results of this paper to richer separation logics (SL) such as SL with nested data structures or SL with limited support for arithmetic reasoning.

## References

[1] T. Antonopoulos, N. Gorogiannis, C. Haase, M. I. Kanovich, and J. Ouaknine. Foundations for decision problems in separation logic with general inductive predicates. In *Foundations of Software Science and Computation Structures - 17th International Conference, FOSSACS 2014, Held as Part of the European Joint Conferences on Theory and Practice of Software, ETAPS 2014, Grenoble, France, April 5–13, 2014, Proceedings*, pages 411–425. Springer, Berlin Heidelberg, 2014.

[2] A. W. Appel. *Program Logics - for Certified Compilers*. Cambridge University Press, 2014.

[3] J. Berdine, C. Calcagno, and P. W. O’Hearn. A decidable fragment of separation logic. In *FSTTCS 2004: Foundations of Software Technology and Theoretical Computer Science, 24th International Conference, Chennai, India, December 16–18, 2004, Proceedings*, pages 97–109. Springer, 2004.

[4] J. Berdine, C. Calcagno, and P. W. O’Hearn. Symbolic execution with separation logic. In *Programming Languages and Systems, Third Asian Symposium, APLAS 2005, Tsukuba, Japan, November 2–5, 2005, Proceedings*, pages 52–68. 2005.

[5] J. Berdine, B. Cook, and S. Ishtiaq. Slayer: Memory safety for systems-level code. In *Computer Aided Verification - 23rd International Conference, CAV 2011, Snowbird, UT, USA, July 14–20, 2011. Proceedings*, pages 178–183, 2011.

[6] S. Blom and M. Huisman. Witnessing the elimination of magic wands. *International Journal on Software Tools for Technology Transfer*, 17(6), 757–781, 2015. 10.1007/s10009-015-0372-3 PMC4841211 27194940

[7] R. Bornat, C. Calcagno, P. W. O’Hearn, and M. J. Parkinson. Permission accounting in separation logic. In *Proceedings of the 32nd ACM SIGPLAN-SIGACT Symposium on Principles of Programming Languages, POPL 2005, Long Beach, California, USA, January 12–14, 2005*, pages 259–270, 2005.

[8] R. Brochenin, S. Demri, and E. Lozes. On the almighty wand. *Information and Computation*, 211:106–137, 2012.

[9] C. Calcagno, D. Distefano, J. Dubreil, D. Gabi, P. Hooimeijer, M. Luca, P. O’Hearn, I. Papakonstantinou, J. Purbrick, and D. Rodriguez. Moving fast with software verification. In K. Havelund, G. Holzmann, and R. Joshi, editors, *NASA Formal Methods: 7th International Symposium, NFM 2015, Pasadena, CA, USA, April 27–29, 2015, Proceedings*, pages 3–11, Cham, 2015. Springer International Publishing.

[10] C. Calcagno, D. Distefano, P. W. O’Hearn, and H. Yang. Compositional shape analysis by means of bi-abduction. *J. ACM*, 58(6):26:1–26:66, Dec. 2011.

[11] C. Calcagno, P. O’Hearn, and H. Yang. Local action and abstract separation logic. In *Logic in Computer Science, 2007. LICS 2007. 22nd Annual IEEE Symposium on*, pages 366–378, July 2007.

[12] C. Calcagno, H. Yang, and P. O’Hearn. Computability and complexity results for a spatial assertion language for data structures. In R. Hariharan, V. Vinay, and M. Mukund, editors, *FST TCS 2001: Foundations of Software Technology and Theoretical Computer Science*, volume 2245 of *Lecture Notes in Computer Science*, pages 108–119. Springer, Berlin Heidelberg, 2001.

[13] B. Cook, C. Haase, J. Ouaknine, M. J. Parkinson, and J. Worrell. Tractable reasoning in a fragment of separation logic. In *CONCUR 2011 - Concurrency Theory - 22nd International Conference, CONCUR 2011, Aachen, Germany, September 6–9, 2011. Proceedings*, pages 235–249, 2011.

[14] S. Demri and M. Deters. Expressive completeness of separation logic with two variables and no separating conjunction. In *Proceedings of the Joint Meeting of the Twenty-Third EACSL Annual Conference on Computer Science Logic (CSL) and the Twenty-Ninth Annual ACM/IEEE Symposium on Logic in Computer Science (LICS)*, CSL-LICS ’14, pages 37:1–37:10, New York, NY, USA, 2014. ACM.

[15] S. Demri, D. Galmiche, D. Larchey-Wendling, and D. Méry. Separation logic with one quantified variable. In E. Hirsch, S. Kuznetsov, J.-É. Pin, and N. Vereshchagin, editors, *Computer Science - Theory and Applications*, volume 8476 of *Lecture Notes in Computer Science*, pages 125–138. Springer International Publishing, 2014.

[16] S. Demri, É. Lozes, and A. Mansutti. The effects of adding reachability predicates in propositional separation logic. In C. Baier and U. Dal Lago, editors, *Foundations of Software Science and Computation Structures*, pages 476–493, Cham, 2018. Springer International Publishing.

[17] K. Dudka, P. Peringer, and T. Vojnar. Predator: A practical tool for checking manipulation of dynamic data structures using separation logic. In *Computer Aided Verification - 23rd International Conference, CAV 2011, Snowbird, UT, USA, July 14–20, 2011. Proceedings*, pages 372–378, 2011.

[18] M. Echenim, R. Iosif, and N. Peltier. The bernays-schönfinkel-ramsey class of separation logic on arbitrary domains. In M. Bojańczyk and A. Simpson, editors, *Foundations of Software Science and Computation Structures*, pages 242–259, Cham, 2019. Springer International Publishing.

[19] N. Gorogiannis, M. Kanovich, and P. W. O’Hearn. The complexity of abduction for separated heap abstractions. In E. Yahav, editor, *Static Analysis: 18th International Symposium, SAS 2011, Venice, Italy, September 14–16, 2011. Proceedings*, pages 25–42, Berlin, Heidelberg, 2011. Springer, Berlin Heidelberg.

[20] X. Gu, T. Chen, and Z. Wu. A complete decision procedure for linearly compositional separation logic with data constraints. In *Automated Reasoning - 8th International Joint Conference, IJCAR 2016, Coimbra, Portugal, June 27 - July 2, 2016*, Proceedings, pages 532–549, 2016.

[21] R. Iosif, A. Rogalewicz, and J. Simácek. The tree width of separation logic with recursive definitions. In *Automated Deduction - CADE-24 - 24th International Conference on Automated Deduction, Lake Placid, NY, USA, June 9–14, 2013. Proceedings*, pages 21–38, 2013.

[22] R. Iosif, A. Rogalewicz, and T. Vojnar. Deciding entailments in inductive separation logic with tree automata. In *Automated Technology for Verification and Analysis - 12th International Symposium, ATVA 2014, Sydney, NSW, Australia, November 3–7, 2014, Proceedings*, pages 201–218, 2014.

[23] S. S. Ishtiaq and P. W. O’Hearn. BI as an assertion language for mutable data structures. In *Conference Record of POPL 2001: The 28th ACM SIGPLAN-SIGACT Symposium on Principles of Programming Languages, London, UK, January 17–19, 2001*, pages 14–26, 2001.

[24] B. Jacobs, J. Smans, P. Philippaerts, F. Vogels, W. Penninckx, and F. Piessens. Verifast: A powerful, sound, predictable, fast verifier for C and java. In *NASA Formal Methods - Third International Symposium, NFM 2011, Pasadena, CA, USA, April 18–20, 2011. Proceedings*, pages 41–55, 2011.

[25] J. Katelaan, D. Jovanović, and G. Weissenbacher. A separation logic with data: Small models and automation. In D. Galmiche, S. Schulz, and R. Sebastiani, editors, *Automated Reasoning*, pages 455–471, Cham, 2018. Springer International Publishing.

[26] J. Katelaan, C. Matheja, and F. Zuleger. Effective entailment checking for separation logic with inductive definitions. In *Tools and Algorithms for the Construction and Analysis of Systems - 25th International Conference, TACAS 2019, Held as Part of the European Joint Conferences on Theory and Practice of Software, ETAPS 2019, Prague, Czech Republic, April 6–11, 2019, Proceedings, Part II*, pages 319–336, 2019.

[27] J. Katelaan and F. Zuleger. Beyond symbolic heaps: Deciding separation logic with inductive definitions. In E. Albert and L. Kovács, editors, *LPAR 2020: 23rd International Conference on Logic for Programming, Artificial Intelligence and Reasoning, Alicante, Spain, May 22–27, 2020*, volume 73 of *EPiC Series in Computing*, pages 390–408. EasyChair, 2020.

[28] Q. L. Le, M. Tatsuta, J. Sun, and W. Chin. A decidable fragment in separation logic with inductive predicates and arithmetic. In *Computer Aided Verification - 29th International Conference, CAV 2017, Heidelberg, Germany, July 24–28, 2017, Proceedings, Part II*, pages 495–517, 2017.

[29] P. Madhusudan, G. Parlato, and X. Qiu. Decidable logics combining heap structures and data. In *Proceedings of the 38th Annual ACM SIGPLAN-SIGACT Symposium on Principles of Programming Languages*, POPL ’11, pages 611–622, New York, NY, USA, 2011. ACM.

[30] P. W. O’Hearn. Resources, concurrency, and local reasoning. *Theor. Comput. Sci*., 375(1–3):271–307, 2007.

[31] J. Pagel. *Decision Procedures for Separation Logic: Beyond Symbolic Heaps*. PhD thesis, TU Wien, 2020. https://repositum.tuwien.at/handle/20.500.12708/16333.

[32] J. Pagel, C. Matheja, and F. Zuleger. Complete entailment checking for separation logic with inductive definitions. *CoRR*, abs/2002.01202, 2020.

[33] J. Pagel and F. Zuleger. Strong-separation logic. *CoRR*, abs/2001.06235, 2020.

[34] J. A. N. Pérez and A. Rybalchenko. Separation logic modulo theories. In *Programming Languages and Systems - 11th Asian Symposium, APLAS 2013, Melbourne, VIC, Australia, December 9–11, 2013. Proceedings*, pages 90–106. 2013.

[35] R. Piskac, T. Wies, and D. Zufferey. Automating separation logic using SMT. In N. Sharygina and H. Veith, editors, *Computer Aided Verification*, volume 8044 of *Lecture Notes in Computer Science*, pages 773–789. Springer, Berlin Heidelberg, 2013.

[36] R. Piskac, T. Wies, and D. Zufferey. Automating separation logic with trees and data. In *Computer Aided Verification - 26th International Conference, CAV 2014, Held as Part of the Vienna Summer of Logic, VSL 2014, Vienna, Austria, July 18–22, 2014. Proceedings*, pages 711–728, 2014.

[37] X. Qiu, P. Garg, A. Stefanescu, and P. Madhusudan. Natural proofs for structure, data, and separation. In *ACM SIGPLAN Conference on Programming Language Design and Implementation, PLDI ’13, Seattle, WA, USA, June 16–19, 2013*, pages 231–242, 2013.

[38] A. Reynolds, R. Iosif, and C. Serban. Reasoning in the bernays-schönfinkel-ramsey fragment of separation logic. In *Verification, Model Checking, and Abstract Interpretation - 18th International Conference, VMCAI 2017, Paris, France, January 15–17, 2017, Proceedings*, pages 462–482, 2017.

[39] A. Reynolds, R. Iosif, C. Serban, and T. King. A decision procedure for separation logic in smt. In C. Artho, A. Legay, and D. Peled, editors, *Automated Technology for Verification and Analysis*, pages 244–261, Cham, 2016. Springer International Publishing.

[40] J. C. Reynolds. Separation logic: A logic for shared mutable data structures. In *17th IEEE Symposium on Logic in Computer Science (LICS 2002), 22–25 July 2002, Copenhagen, Denmark, Proceedings*, pages 55–74, 2002.

[41] M. Schwerhoff and A. J. Summers. Lightweight support for magic wands in an automatic verifier. In *29th European Conference on Object-Oriented Programming, ECOOP 2015, July 5–10, 2015, Prague, Czech Republic*, pages 614–638, 2015.

[42] M. Tatsuta and D. Kimura. Separation logic with monadic inductive definitions and implicit existentials. In *Programming Languages and Systems - 13th Asian Symposium, APLAS 2015, Pohang, South Korea, November 30 - December 2, 2015, Proceedings*, pages 69–89, 2015.

